# Comparison of three gamma oscillations in the mouse entorhinal–hippocampal system

**DOI:** 10.1111/ejn.13831

**Published:** 2018-02-09

**Authors:** James L. Butler, Y. Audrey Hay, Ole Paulsen

**Affiliations:** ^1^ Physiological Laboratory Department of Physiology, Development and Neuroscience University of Cambridge Downing Street Cambridge CB2 3EG UK

**Keywords:** neural circuit, neural network, optogenetics, theta

## Abstract

The entorhinal–hippocampal system is an important circuit in the brain, essential for certain cognitive tasks such as memory and navigation. Different gamma oscillations occur in this circuit, with the medial entorhinal cortex (mEC), CA3 and CA1 all generating gamma oscillations with different properties. These three gamma oscillations converge within CA1, where much work has gone into trying to isolate them from each other. Here, we compared the gamma generators in the mEC, CA3 and CA1 using optogenetically induced theta–gamma oscillations. Expressing channelrhodopsin‐2 in principal neurons in each of the three regions allowed for the induction of gamma oscillations via sinusoidal blue light stimulation at theta frequency. Recording the oscillations in CA1 *in vivo*, we found that CA3 stimulation induced slower gamma oscillations than CA1 stimulation, matching *in vivo* reports of spontaneous CA3 and CA1 gamma oscillations. In brain slices *ex vivo*, optogenetic stimulation of CA3 induced slower gamma oscillations than stimulation of either mEC or CA1, whose gamma oscillations were of similar frequency. All three gamma oscillations had a current sink–source pair between the perisomatic and dendritic layers of the same region. Taking advantage of this model to analyse gamma frequency mechanisms in slice, we showed using pharmacology that all three gamma oscillations were dependent on the same types of synaptic receptor, being abolished by blockade of either type A γ‐aminobutyric acid receptors or α‐amino‐3‐hydroxy‐5‐methyl‐4‐isoxazolepropionic acid/kainate receptors, and insensitive to blockade of *N*‐methyl‐d‐aspartate receptors. These results indicate that a fast excitatory–inhibitory feedback loop underlies the generation of gamma oscillations in all three regions.

## Introduction

The entorhinal–hippocampal system is an important circuit in the brain, essential for some cognitive functions such as memory and navigation. This circuit involves a reciprocal processing loop consisting of the medial entorhinal cortex (mEC), and cornu ammonis (CA) 1 and CA3 of the hippocampus proper. Gamma oscillations are an emergent property of this circuit, with a variety of gamma oscillations being observed across the different regions *in vivo*. A slow gamma oscillation with frequency ranging between 30 and 80 Hz is generated in CA3 and propagates to the downstream CA1 (Csicsvari *et al*., 2003). A medium frequency gamma oscillation, in the range of 60–120 Hz, is generated in the mEC and also propagates to CA1 (Colgin *et al*., 2009). More recently, a third locally generated faster gamma oscillation (> 100 Hz) was observed in CA1 (Belluscio *et al*., 2012; Schomburg *et al*., 2014; Lasztóczi & Klausberger, 2016). The exact properties of these three different gamma oscillations, and how they either differ from or are similar to oscillations generated in the other regions, are currently unclear.

A variety of models has been developed for generating gamma oscillations in *ex vivo* slices from the entorhinal cortex and hippocampus, and these have been used to analyse the properties of the individual gamma generators. Two main mechanisms have been suggested to be responsible for gamma oscillations generation and maintenance. In the pyramidal‐interneuron network gamma (PING) model, excitation of pyramidal neurons causes the local interneurons they innervate to fire, which then feedback inhibits pyramidal neurons until the inhibition fades and the next cycle can occur (Fisahn *et al*., 1998; Whittington *et al*., 2000; Mann *et al*., 2005). Alternatively, the interneuron network gamma (ING) model proposes that tonic excitation of interneurons, synchronised among themselves, causes them to fire at their preferred firing frequency, resulting in rhythmic synchronous inhibition of the entire network and therefore the length of each gamma cycle corresponds to the interspike interval of the interneurons (Bartos *et al*., 2007). In CA3 or mEC slices, application of respectively the acetylcholine receptor agonist carbachol or kainate causes the generation of gamma oscillations (Fisahn *et al*., 1998; Cunningham *et al*., 2003), which are both generated and maintained through a PING mechanism (Fisahn *et al*., 1998; Whittington *et al*., 2000; Mann *et al*., 2005). In CA1, both ING (Whittington *et al*., 1995; Craig & McBain, 2015) and PING mechanisms (Pietersen *et al*., 2014; Butler *et al*., 2016) have been described to sustain gamma oscillations.

More recently, the advent of optogenetics has opened up new research avenues into gamma oscillations. It has been demonstrated that by stimulating the optogenetic activator channelrhodopsin‐2 (ChR2), expressed either in excitatory neurons of CA3 or CA1, or in stellate cells and interneurons in the mEC, local gamma oscillations can be induced in each of these areas (Akam *et al*., 2012; Pastoll *et al*., 2013; Butler *et al*., 2016; Betterton *et al*., 2017). These *ex vivo* optogenetically induced gamma oscillations are similar to gamma oscillations seen *in vivo* and provide a precise way with which to induce gamma oscillations in the local region.

Combining optogenetic stimulation and multielectrode array (MEA) recordings, we aimed to compare the gamma generators in the mEC, CA3 and CA1. Using transgenic mouse lines to express ChR2 in principal neurons of the mEC, CA3 and CA1, robust gamma oscillations were induced with theta frequency sinusoidal blue light stimulation. In agreement with *in vivo* observations, CA3 gamma oscillations in slice were slower than those in the mEC and CA1, but there was no discernible difference in the frequency of the mEC and CA1 gamma oscillations. A two‐dimensional current source density analysis showed that all three regions had a current sink–source pair between the perisomatic and dendritic layers of the region. All three gamma oscillations were dependent on the same types of synaptic receptor, being abolished by blockade of either type A γ‐aminobutyric acid (GABA_A_) receptors or AMPA/kainate receptors, and insensitive to blockade of NMDA receptors.

## Methods

### Mice

**Figure 1:**
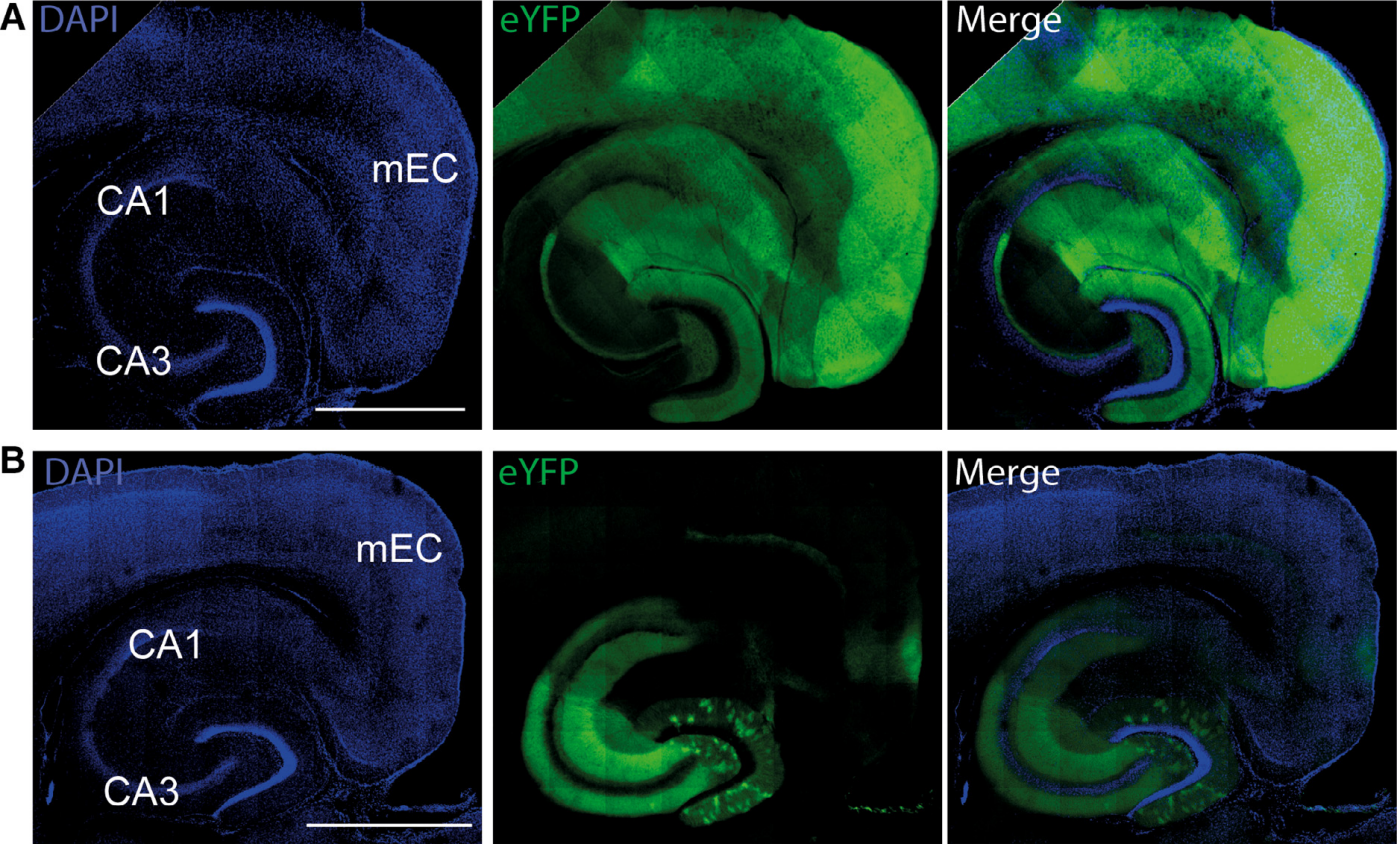
Channelrhodopsin‐2 expression in acute hippocampal slices. (A) The F1 generation from CaMKIIα‐Cre X ChR2‐eYFP had strong enhanced yellow fluorescent protein (eYFP) expression in CA1, the dentate gyrus and the cortex, but not in CA3. The left panel shows the staining for the DNA‐binding fluorescent marker, DAPI. The middle panel shows the eYFP expression, which is indicative of ChR2 expression, and the right panel is the overlay of the left and middle panels. Scale bar, 1 mm. (B) The eYFP expression in the F1 generation from Grik4‐Cre X ChR2‐eYFP was limited to the CA3 region and the layers of CA1 that CA3 principal neurons project to in addition to sparse expression in the dentate gyrus.

Three different transgenic lines were used for the experiments, all of which were maintained as homozygous colonies. Two Cre lines were used, +/+ CaMKII‐α‐Cre mice on a C57BL/6 background (Jackson Laboratories, Maine, USA, stock #005359; http://jaxmice.jax.org/strain/005359.html) and +/+ Grik4‐Cre mice on a C57BL/6 background (Jackson Laboratories, stock #006474; http://www.jax.org/strain/006474) to express Cre in principal neurons of the mEC and CA1, and in CA3, respectively. These were both crossed with +/+ LoxP‐ChR2(H134R)‐EYFP mice on a 129S6 background (Jackson Laboratories, stock #012569; http://jaxmice.jax.org/strain/012569.html), to generate transgenic mice expressing ChR2 in principal neurons in either the mEC and CA1 (herein referred to as CaMKIIα‐ChR2 mice, Fig. 1A) or the CA3 (herein referred to as Grik4‐ChR2 mice, Fig. 1B). Only first generation offspring aged between 4 and 8 weeks of both genders were used in experiments. The research was performed under the Animals (Scientific Procedures) Act 1986 Amendment Regulations 2012 following ethical review by the University of Cambridge Animal Welfare and Ethical Review Body (AWERB). The animal procedures were authorised under Personal and Project licences held by the authors.

### *In vivo* recordings

Mice were anaesthetised with 2 g/kg urethane (Sigma‐Aldrich, Missouri, USA) diluted 20% w/v in saline. This sedation was supplemented with 0.2 g/kg urethane as needed throughout the experiment (approximately once every 2 h). Mice were left in a dark, quiet, heated environment while the anaesthetic took effect. This was defined as the point at which the animal no longer had any paw pinch or blink reflexes, typically taking up to 1 h 15 min. The following coordinates from bregma were then used to target the CA1 region: anterior/posterior: −1.94 mm, medial/lateral: 1.75 mm, dorsal/ventral: −1.2 mm. A small hole approximately 0.5 mm in diameter was then drilled at the appropriate position using a micro drill (CellPoint Scientific, Maryland, USA). The surface of the exposed brain was covered with saline (0.9% NaCl) for the duration of the experiment.

To record the local field potential (LFP) *in vivo*, a 2 MΩ tungsten recording electrode (A‐M systems, Hinckley, United Kingdom (UK)) was attached to a 200 μm diameter optical fibre (Thorlabs, New Jersey, USA) with the electrode tip protruding approximately 100 μm past the tip of the optical fibre. This optrode was slowly lowered into the CA1 *stratum pyramidale* of the hippocampus, and a 473 nm diode‐pumped solid‐state laser (Ciel, Laser Quantum, Cheshire, UK) was used to deliver blue light through the optic fibre, at an intensity of up to 5 mW/mm^2^. A two‐channel amplifier (Microelectrode AC Amplifier Model 1800, A‐M systems) was used to record LFP from the electrode at an acquisition rate of 20 kHz. Data were acquired using an ITC18 acquisition data board (HEKA Instruments, Lambrecht/Pfalz, Germany) and using custom written procedures in Igor Pro (WaveMetrics, Oregon, USA). After a recording session was complete, a 3 mA current was passed through the recording electrode for 2 s, lesioning the recording site. Hippocampal slices were then prepared as described below, and the lesion site was identified under a bright‐field microscope to confirm recording location.

### Slice preparation

Mice were deeply anaesthetised with 4% (v/v) isoflurane at 1.4 L/min and decapitated. The brain was then quickly excised and horizontal slices 400 μm in thickness were prepared in cold (0–3 °C), oxygenated (95% O_2_, 5% CO_2_) artificial cerebrospinal fluid (aCSF) containing: 126 mm NaCl, 3 mm KCl, 1.25 mm NaH_2_PO_4_, 2 mm MgSO_4_, 2 mm CaCl_2_, 26.4 mm NaHCO_3_ and 10 mm glucose (pH 7.2). Slices were stored in a submerged‐style storage chamber at room temperature (22–24 °C) for at least 50 min prior to use.

### Multielectrode recordings

All LFP recordings were conducted at a 20 kHz acquisition rate using the Panasonic MED64 system (Alpha MED Scientific Inc, Osaka, Japan). All recordings were performed on 64‐channel probes, whose electrodes measured 50 × 50 μm and were positioned in an even 8 × 8 grid spanning 1 mm^2^ (Panasonic MED‐P5155; Tensor Biosciences, Irvine, CA, USA). These probes were coated in poly‐d‐lysine before use to aid with slice adhesion. They were then rinsed with aCSF and a slice positioned on the probe to incorporate both the dendritic and perisomatic layers of the region of interest. Slices were then maintained in a submerged condition, continually superfused with aCSF at a rate of 3–4 mL/min and heated to 27–29 °C. The slices were left for at least 10 min before any recording commenced.

### Light delivery

Channelrhodopsin‐2 was excited using either a 470 nm digital micromirror device (Polygon, Mightex, Sussex, UK) or a 473 nm diode‐pumped solid‐state laser (Ciel, Laser Quantum, Cheshire, UK). An Olympus BX51 microscope was used to deliver the light to the slices through a 10× objective, which caused illumination of a 500 μm diameter circle or a 500 × 500 μm square in the case of the laser and digital micromirror device, respectively. The centre of the light was positioned over the perisomatic layer of the region in question, resulting in illumination of all of the perisomatic layer and also the neighbouring dendritic layers each time. Custom made stimulation protocols executed in Igor Pro (WaveMetrics, Oregon, USA) were used to send control signals to both the light sources and the recording software, allowing for synchronisation of the light with the LFP recordings. The maximum light intensity (100%) was less than 2.5 mW/mm^2^ in all cases, and the waveform of the optical stimulation was normally a 5 Hz sinusoidal curve varying from a minimum of 0 mW/mm^2^ to a maximum of 2.5 mW/mm^2^.

### Imaging

Channelrhodopsin‐2 expression was assessed using a mercury lamp to excite eYFP, and the emission observed using a 540 nm filter fitted into an Olympus BX51 microscope. A subset of slices was fixed overnight using a 4% paraformaldehyde solution. The slices were then washed in phosphate‐buffered saline (PBS) solution, incubated in DAPI for no more than 5 min and then fixed on a microscope slide. The slides were left at 4 °C in darkness for at least 24 h before being imaged. A Leica SP2 confocal microscope was used to image the slices using excitation wavelengths of 405 and 514 nm, and emission wavelengths of 420–460 nm and 530–560 nm, for DAPI and eYFP, respectively.

For parvalbumin (PV) and GFP co‐labelling, 6‐ to 8‐week‐old mice were perfused transcardially using PBS solution containing 4% paraformaldehyde. Brains were kept overnight in 4% paraformaldehyde for better fixation and then were cryoprotected using 30% sucrose solution. Brains were sliced to 80 μm thickness using a freezing microtome and kept in PBS at 4 °C. Free‐floating sections were blocked for 2 h at room temperature in a PBS/0.25% Triton X‐100/0.2% gelatine solution (PBS‐GT) before being incubated overnight at room temperature with Rabbit anti‐PV (1/2000; Abcam ab11427) and Chicken anti‐GFP (1/2000; Abcam ab13970) primary antibodies diluted in PBS‐GT. After extensive washing in PBS‐GT, slices were incubated for 3 h at room temperature with Goat anti‐Chicken Alexafluor488 (1/400; Life Technologies A11039) and Donkey anti‐Rabbit Alexafluor568 (1/400; Life Technologies A10042) secondary antibodies diluted in PBS‐GT. After several washes in PBS sections were mounted in Fluoroshield media. Fluorescent images were acquired with a scanning confocal microscope (SP8; Leica) using 488 and 561 nm lasers and 63× magnification objective. Counting was performed using the imagej software.

### Pharmacology

All drugs and reagents were purchased from either Sigma‐Aldrich (Poole, UK) or Tocris (Bristol, UK). Drugs were prepared as stock solutions 1000× the desired concentration using the following solvents: dimethyl sulfoxide (DMSO) for the GABA_A_ receptor antagonist (+)‐bicuculline and the α‐amino‐3‐hydroxy‐5‐methyl‐4‐isoxazolepropionic acid (AMPA) and kainate receptor antagonist 2,3‐dihydroxy‐6‐nitro‐7‐sulfamoyl‐benzo[f]quinoxaline‐2,3‐dione (NBQX); H_2_0 for the *N*‐methyl‐d‐aspartate (NMDA) receptor antagonist dl‐2‐amino‐5‐phosphonopentanoic acid (AP5). These stock solutions were then frozen and defrosted on the morning of the relevant experiment, at which time they were diluted 1000× in aCSF. After a 10‐min incubation period, a recording of activity was taken during stimulation with blue light. The relevant drug solution was then bath applied and another recording taken 10 min later.

### Data analysis and statistics

All analysis was conducted in Igor Pro (WaveMetrics, OR, USA) using custom written protocols. Data were first imported, downsampled to a 2000 Hz sampling rate and band‐pass filtered between 20 and 120 Hz using a finite response filter. All 64 channels were then analysed using Welch's power spectral density (PSD); from which the area under the PSD ± 15 Hz from the peak in the gamma range (30–100 Hz) was used to determine the power of the gamma oscillations. The channel with the highest power from each recording was selected for further analysis. If the peak was at a harmonic of theta oscillations at 30 Hz or below, these cases were excluded from further analysis.

Continuous wavelet transforms were calculated using a normalised Morlet wavelet transform (ω_0_ = 8). This was performed for each individual theta cycle, and the resulting scalograms were then averaged.

A current source density (CSD) profile was constructed from signals recorded on the 64‐channel array as described previously (Mann *et al*., 2005; Butler *et al*., 2016). Briefly, cycle averages were calculated for each channel from 48 consecutive theta cycles. These averages were band‐pass filtered between 30 and 120 Hz to exclude harmonics of the theta oscillations caused by the light stimulation. Next, the averages were smoothed using a 3 × 3 Gaussian filter and convolved with a 3 × 3 Laplacian filter (0 −1 0, −1 4 −1, 0 −1 0) to attain the second spatial derivative. To avoid edge artefacts signals from the outer 28 electrodes are not presented.

To characterise the temporal waveform of the oscillation, an asymmetry index was defined as the duration of the descending phase as a fraction of a half cycle, −1. This index produces a value of 0 for a perfectly symmetric waveform, a value between 0 and 1 for waveforms with a shorter ascending phase (with 1 being if the descending phase occupied the entire waveform) and between 0 and −1 for waveforms with a longer ascending phase.

In the pharmacology experiments, there was a small rundown of the power of the gamma oscillations during the 10‐min incubation period when no drug was added. Therefore, the change in gamma oscillations caused by drugs was compared to the change observed when no drug was added. Differences were statistically assessed using an independent samples two‐tailed Student's *t*‐test. All averages presented are mean ± standard error of the mean, except for circular data in which case the circular mean and accompanying *r* value are presented.

## Results

### Optogenetic gamma oscillations in the entorhinal–hippocampal circuit

Crossing transgenic mouse lines expressing Cre recombinase in principal neurons with a transgenic mouse line harbouring a LoxP‐flanked ChR2 gene allowed for specific targeting of ChR2. The ChR2 was fused to eYFP allowing for visualisation of the ChR2 expression using fluorescence microscopy (Fig. 1). The CaMKIIα‐specific ChR2 showed strong eYFP expression in CA1, the dentate gyrus and the cortex, but no obvious expression in CA3 (Fig. 1A). Expression in the Grik4‐specific ChR2 was almost inverse of this, with strong eYFP expression evident in the CA3 region, sparse expression in the dentate gyrus and no observable expression elsewhere (Fig. 1B). Therefore, using the mice from the two crosses, acute slices could be produced that expressed ChR2 in either the mEC and CA1, or the CA3.

It has been reported that some interneurons also express CaMKIIα, and, as interneurons play an important role in the generation of gamma oscillations, we quantified the percentage of PV‐positive interneurons expressing ChR2 in the CA3 of Grik4‐ChR2 and in the CA1 of CaMKIIα‐ChR2 mice. We observed that 13.8% of PV cells expressed ChR2 in CA3 of Grik4‐ChR2 mice (*n* = 99 cells from two animals), and 12.5% in CA1 of CaMKIIα‐ChR2 mice (*n* = 72 cells from *n* = 4 animals). Therefore, a minor population of PV interneurons of equivalent size would be activated by light stimulation in both transgenic models.

**Figure 2:**
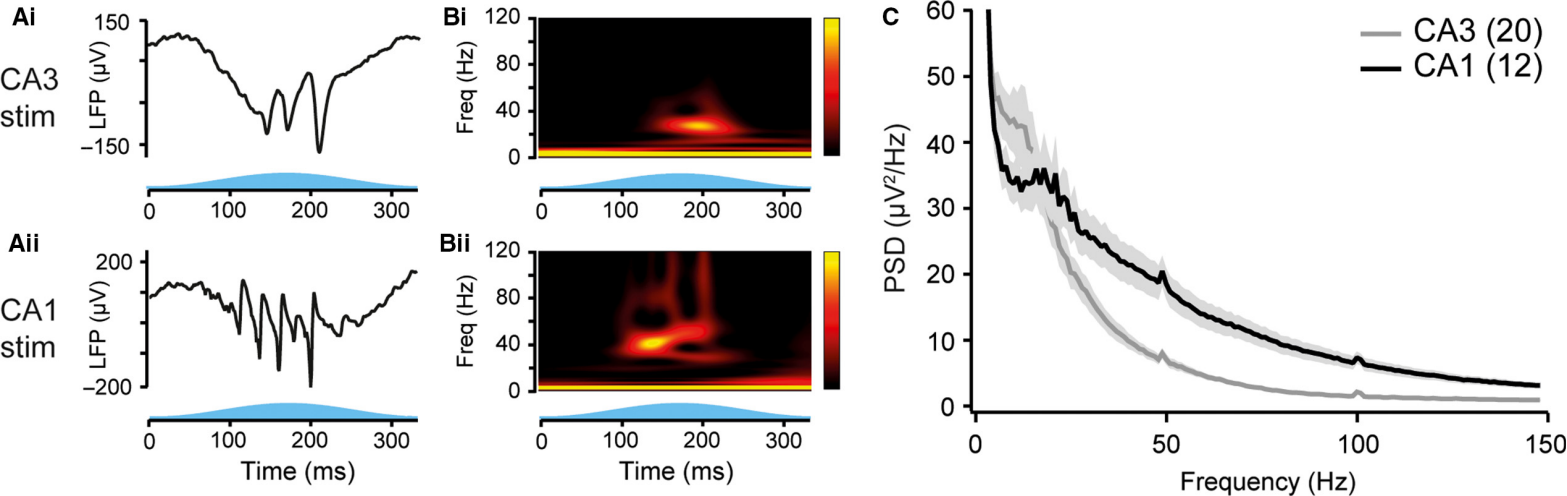
Optogenetic induction of gamma oscillations *in vivo*. (A) Representative trace of local field potential (LFP) activity (top) recorded from the perisomatic layer of CA1 during sinusoidally modulated theta frequency blue light stimulation of either CA3 (i) or CA1 (ii) pyramidal neurons. (B) Continuous wavelet transform of the recordings shown in A. Freq, frequency. (C) Average power spectral density plots of CA1 LFP recordings taken during either CA3 (grey) or CA1 (black) stimulation. Shaded regions represent standard error of the mean.

Adult mice of both genotypes were anaesthetised with 2 g/kg urethane. Urethane is known to attenuate input to the CA1 from the EC (Ylinen *et al*., 1995), therefore allowing the study of CA3 and CA1 gamma oscillations during reduced mEC activity. An electrode with an optical fibre attached was lowered into the pyramidal layer of CA1 and blue light delivered, sinusoidally modulated at a frequency of 3 Hz. Stimulation of either the CA1 pyramidal neurons in CaMKIIα‐ChR2 or stimulation of the CA3 pyramidal neurons in Grik4‐ChR2 mice induced gamma oscillations in the stratum pyramidale of CA1 (Fig. 2A). Both gamma oscillations were nested in the theta oscillations, occurring at the peak of light stimulation, or the trough of the LFP theta oscillation as recorded from the perisomatic layer (Fig. 2B). The frequency of the gamma oscillations differed between activation of the two regions, with a shift to lower frequencies for CA3 stimulation compared to CA1 stimulation [ratio of slow (20–50 Hz) to total gamma power (20–120 Hz) was 0.565 ± 0.012 for CA3 stimulation, *n* = 20 and 0.401 ± 0.001 for CA1 stimulation, *n* = 12; *P* < 0.001, Student's *t*‐test; Fig. 2C]. This is consistent with recordings of naturally occurring gamma oscillations in the CA1 region, which are normally nested within theta oscillations and of higher frequency when generated within CA1 rather than CA3 (Schomburg *et al*., 2014; Lasztóczi & Klausberger, 2016). Optogenetic stimulation of pyramidal neurons can therefore be used as a physiological model to study gamma oscillations.

**Figure 3:**
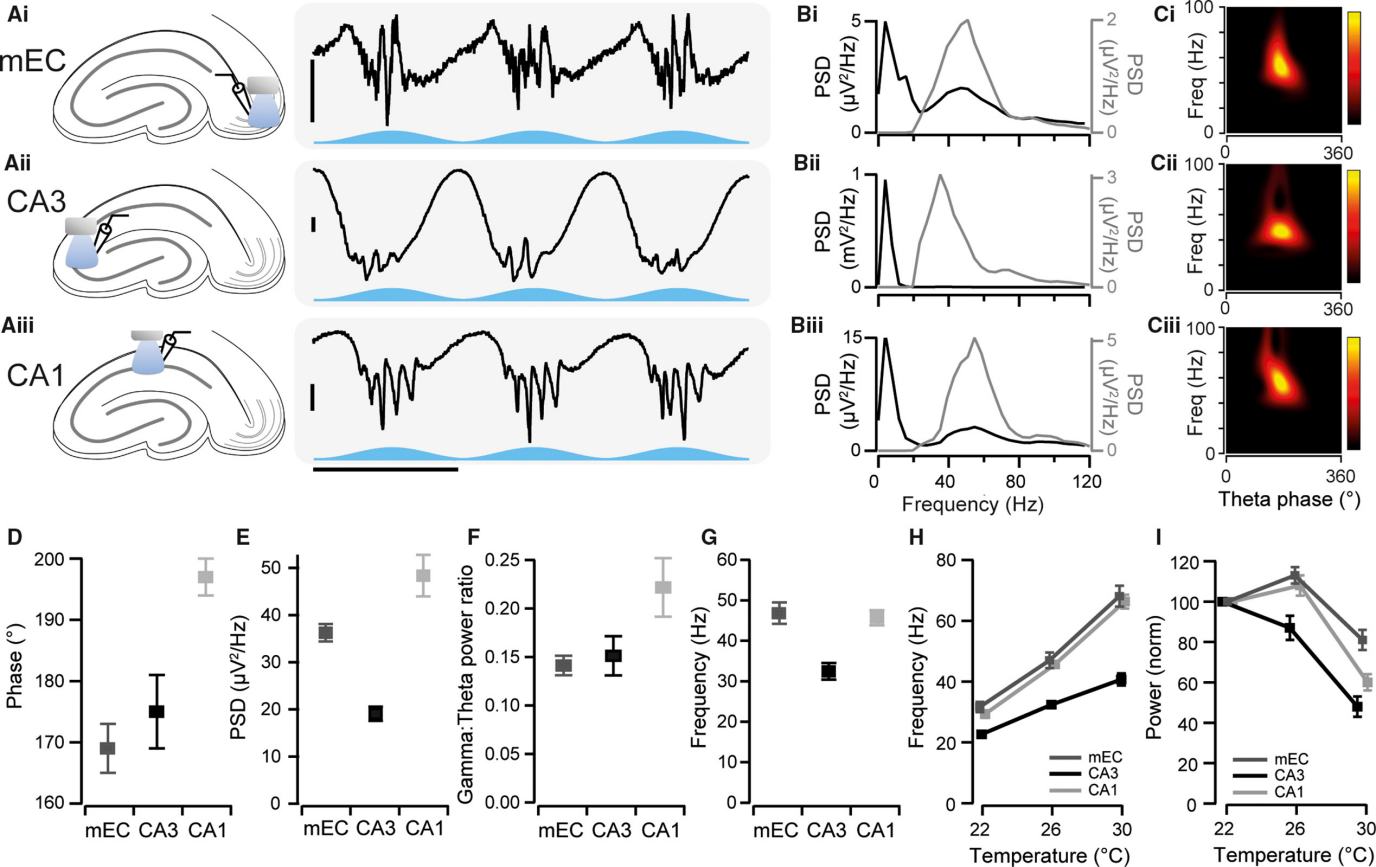
Optogenetic induction of theta‐nested gamma oscillations *ex vivo*. (A) Representative traces of extracellular field activity recorded in the medial entorhinal cortex (mEC, top), CA3 (middle) and CA1 (bottom) during blue light theta stimulation. Horizontal scale bar, 200 ms; vertical scale bar, 50 μV. Blue traces represent the optical stimulation. (B) Power spectral density (PSD) for the entire 10‐second recordings that correspond to the examples shown in A. The black line is the PSD for the widepass‐filtered trace, and grey is the PSD after a 30–120 Hz band‐pass filter was applied to the recordings. (C) Continuous wavelet transforms of recordings as shown in A. Peaks in the theta range were used to divide the recordings into individual theta cycles and the average scalogram of all theta cycles extracted (*n* = 50). (D) Average phase of the theta oscillation at which the peak gamma oscillation occurred in each of the three regions. 0° corresponds to the peak of the theta cycle. (E) Average PSD of the gamma oscillations in the three different regions. (F) Average ratio of the gamma power to the theta power elicited by stimulation. (G) Average peak frequency of gamma oscillations in the three different regions. (H) Frequency of optogenetic gamma oscillations recorded from the dendritic region of CA3 (black, *n* = 14), and from the perisomatic regions of mEC (medium grey, *n* = 12) and CA1 (light grey, *n* = 12) at different temperatures. (I) Average change in the power of the gamma oscillations at different temperatures. Values are normalised to the power at room temperature. (D–I) Error bars represent standard error of the mean.

To allow us to study the gamma oscillation mechanism, we prepared *ex vivo* slices from the CaMKIIα‐ChR2 and Grik4‐ChR2 mice. When blue light, modulated by a 5 Hz sinusoidal wave, was shone on these slices, it induced robust gamma oscillations in all three of the regions of interest (Fig. 3A). All three gamma oscillations had a narrow peak in the gamma range (Fig. 3B), suggesting the activation of a single generator in each region. As is the case with gamma oscillations recorded *in vivo* (Bragin *et al*., 1995; Csicsvari *et al*., 2003 *;* Colgin *et al*., 2009)*,* the gamma oscillations recorded here were all phase‐amplitude coupled to the theta oscillation, having the highest power at the trough of the theta LFP as recorded in the perisomatic region (Fig. 3C). On average, all three gamma oscillations occurred near the trough of the theta LFP oscillation (mEC: 169 ± 4°, *n* = 41; CA3: 175 ± 6°, *n* = 22; CA1: 197 ± 3°, *n* = 36; Fig. 3D). CA1 gamma oscillations had the highest power (113 ± 10 μV^2^/Hz), followed by mEC gamma (78 ± 5 μV^2^/Hz), and CA3 produced gamma oscillations with the lowest power (50 ± 5 μV^2^/Hz, Fig. 3E). The CA1 generator also had a larger gamma/theta power ratio (0.22 ± 0.03 compared to the 0.14 ± 0.01 and 0.15 ± 0.02 for the mEC and CA3, respectively, Fig. 3F).

The traditional way in which the entorhinal–hippocampal gamma oscillations have been classified is by their frequencies. In accordance with *in vivo* observations, CA3 gamma oscillations were found to be the slowest with a frequency of 32 ± 1 Hz at 27–29 °C (*n* = 14). There was little difference between mEC and CA1 gamma oscillations, however, which had frequencies of 47 ± 3 and 46 ± 1 Hz, respectively (*n* = 12 in both cases, Fig. 3G).

As these oscillations were recorded at a lower than physiological temperature, we tested the effect that temperature had on the gamma oscillations. Increasing the temperature caused an increase in the frequency of the oscillations in all three regions. For CA3, gamma oscillations increased from 23 ± 0 Hz at 22 °C to 41 ± 2 Hz at 30 °C (*n* = 14; Fig. 3H), getting closer to the *in vivo* slow gamma frequency (Schomburg *et al*., 2014; Hsiao *et al*., 2016; Lasztóczi & Klausberger, 2016). The frequency of gamma oscillations in the mEC at 30 °C was also in agreement with medium gamma frequency described *in vivo* (Colgin *et al*., 2009; Belluscio *et al*., 2012; Schomburg *et al*., 2014), reaching a frequency of 68 ± 3 Hz at 30 °C (*n* = 12). Gamma oscillations in CA1 had a frequency of 66 ± 2 Hz at 30 °C (*n* = 12; Fig. 3H), slower than fast gamma frequency recorded in rats *in vivo* (Colgin *et al*., 2009; Belluscio *et al*., 2012; Schomburg *et al*., 2014) but comparable to fast gamma oscillations in mice (Mably *et al*., 2017). During these increases in temperature and frequencies, the power of the oscillations decreased across this range (mEC: 19 ± 5% decrease, *n* = 12; CA3: 52 ± 5% decrease, *n* = 14; CA1: 40 ± 4% decrease, *n* = 12; Fig. 3I). Despite the large changes in frequency, the waveforms of the oscillations recorded from the perisomatic regions of the mEC remained consistent (data not shown), suggesting the same mechanism of generation across the temperatures tested. As such, all the subsequent experiments were carried out at 27–29 °C.

**Figure 4:**
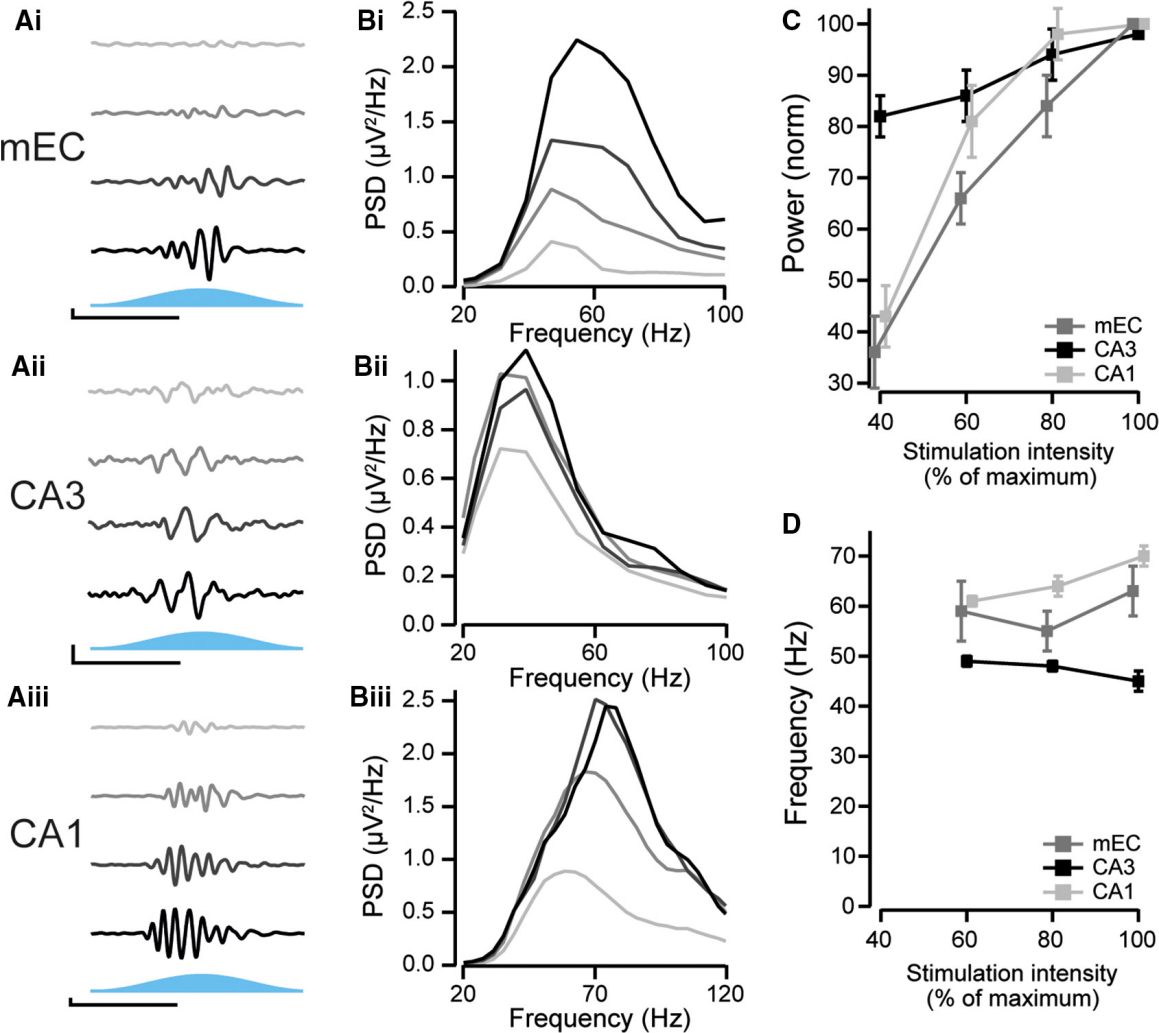
Effect of light intensity on gamma oscillations. (A) Representative traces of medial entorhinal cortex (mEC) (Ai), CA3 (Aii) and CA1 (Aiii) gamma oscillations as stimulation (light) intensity was varied between 40 (top) and 100% (bottom) of maximum in 20% increments. For illustrative purposes, the mEC and CA1 recordings have been high‐pass filtered at 30 Hz and the CA3 recordings at 20 Hz. (B) Power spectral density of the filtered recordings shown in A. (C) Average change in the power of gamma oscillations measured from the perisomatic regions of CA1 and mEC and the dendritic region of CA3 as a function of stimulation intensity (*n* = 6 for CA3, *n* = 10 for mEC,* n* = 11 for CA1). (D) Same as in C but the frequency is displayed. Frequency values could not be reliably estimated for 40% light intensity due to low signal‐to‐noise ratio.

Theta oscillations vary in power and frequency depending on the behavioural state of the animal (McFarland *et al*., 1975; Sławińska & Kasicki, 1998). We therefore examined the effect of changes in stimulation intensity on the gamma oscillations. The maximum intensity of the blue light stimulation was varied between 40 and 100% of the maximum laser power (approximately 2.5 mW/mm^2^) in 20% increments (Fig. 4). The power of the gamma oscillations recorded from all three regions was highly dependent on the strength of theta input, with 40% light intensity producing oscillations with a power of 36 ± 7% (*n* = 9), 82 ± 9% (*n* = 6) and 43 ± 6% (*n* = 11) compared to their power at maximum light intensity for the mEC, CA3 and CA1, respectively (Fig. 4C). Because the gamma power at maximum light intensity was lower in the CA3 region, the magnitude of change is not directly comparable between the regions. Frequency could not be accurately estimated at 40% maximum light intensity due to the lower power of gamma oscillations induced; we therefore compared the frequency at 60, 80 and 100% of maximum light intensity. CA1 gamma oscillation frequency increased significantly from 61 ± 1 to 70 ± 2 Hz (*P* = 0.004, Fig. 4D) when the light intensity was increased from 60 to 100% of maximum intensity. The frequency of mEC and CA3 gamma oscillations, however, was not significantly different between these two light intensities (59 ± 6 and 63 ± 5 Hz, *P* = 0.35, for the mEC, 49 ± 1 and 45 ± 2 Hz, *P* = 0.10, for CA3, Fig. 4D). The different gamma oscillations induced here are therefore very similar to the different gamma oscillations seen in the entorhinal–hippocampal system *in vivo*. ChR2‐mediated stimulation of principal neurons in the entorhinal–hippocampal system can thus be used as an *ex vivo* model of gamma oscillations. We next turned our attention to the underlying mechanisms that gave rise to these oscillations in the three different regions.

### Spatial profile of optogenetic entorhinal–hippocampal gamma oscillations

**Figure 5:**
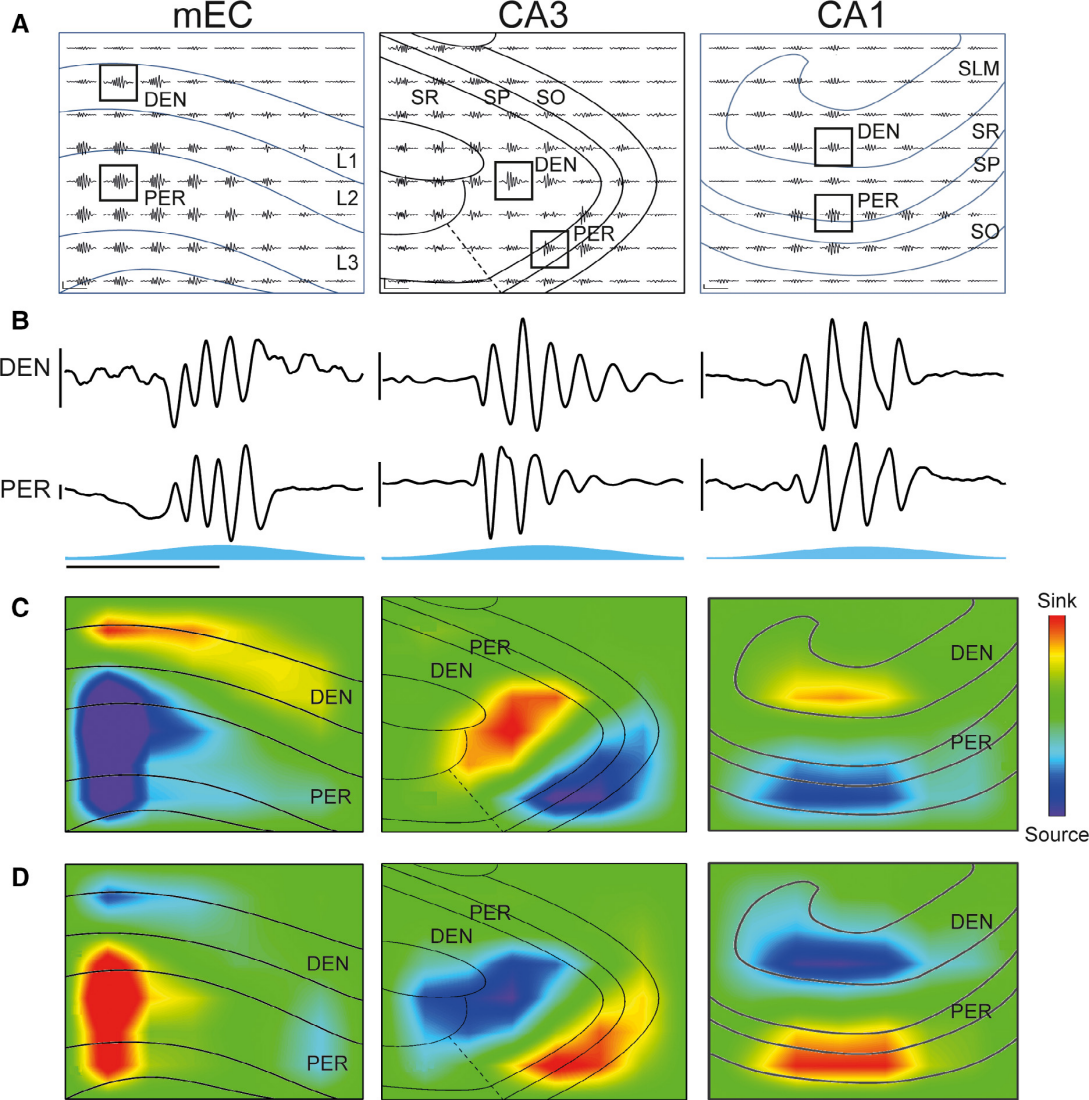
Current source density profile of optogenetic gamma oscillations. (A) Example local field potential recordings taken using a multielectrode array during blue light stimulation of medial entorhinal cortex (mEC) (left), CA3 (middle) and CA1 (right). The average theta‐nested gamma cycle (*n* = 48) for the two channels highlighted with squares is shown in B. Horizontal scale bar, 100 ms; vertical scale bar, 20 μV (left), 10 μV (centre), 50 μV (right). PER, perisomatic layer; DEN, dendritic layer(s); SP, stratum pyramidale; SO, stratum oriens; SR, stratum radiatum; SLM, stratum lacunosum‐moleculare; L1–3, layer 1–3. (B) Enlarged representative traces that are highlighted with black squares in A. Horizontal scale bar, 100 ms; vertical scale bar, 20 μV. (C) Two‐dimensional current source density profile calculated for the 36 inner electrodes shown in A for the trough of a gamma cycle recorded from the perisomatic layer of each respective region. (D) Same as in C but for the peak of a gamma cycle recorded from the perisomatic layer of each respective region. This spatial profile with alternating sink–source pairs was consistent across all slices tested (*n* = 2, 3 and 6 for mEC, CA3 and CA1, respectively).

Gamma oscillations *in vivo* can be recorded in multiple layers simultaneously, with gamma oscillations from different sources residing within different layers of the CA1 region (Belluscio *et al*., 2012; Schomburg *et al*., 2014; Lasztóczi & Klausberger, 2016). As recordings were taken using a 64‐channel multielectrode array, this allowed for simultaneous recording of the LFP in all layers of the local region simultaneously (Fig. 5A). A 2D CSD analysis was used to more accurately localise the current sinks and sources for the different gamma oscillations. The oscillations in all three regions were found to spread outwards by approximately 400 μm from their focal point, decreasing in amplitude with distance from the epicentre (Fig. 5A). As this was indiscriminate of the surrounding areas, with no directional preference to the spread, this was likely due to volume conduction.

In all three regions, gamma oscillations occurred in the perisomatic layer containing the principal neuron cell bodies (stratum pyramidale in CA1 and CA3, and layers II–VI in the mEC). In addition to this, the gamma oscillations also occurred in the layers containing the dendrites of the principal neurons (Fig. 5A and B). Cycle averages were constructed from 48 stimulation theta cycles (Fig. 5B) and in all cases strong gamma peaks persisted, demonstrating the highly regular timing of the gamma cycles between each theta cycle.

The CSD profile revealed alternating sink–source pairs within each local region. In the mEC, the sink–source pair was between layer I and the deeper layers (layers II–V, Fig. 5C and D, left). In CA3, the pairs were between the adjacent stratum pyramidale and stratum radiatum (Fig. 5C and D, centre). In CA1, the pairs were between stratum pyramidale and stratum lacunosum‐moleculare, with no apparent activity in the intermediary stratum radiatum (Fig. 5C and D, right). This was consistent across all recordings tested (*n* = 2, 3 and 6 for mEC, CA3 and CA1, respectively). All three generators therefore showed a reversal in the gamma oscillations between the perisomatic and dendritic regions of their local area, thus demonstrating the local generation of the gamma oscillations.

**Figure 6:**
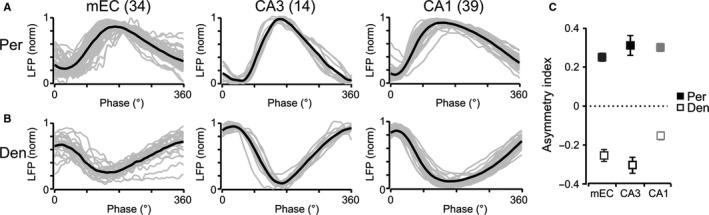
Asymmetry of gamma oscillation waveforms. (A, B) Normalised average gamma cycle for each individual slice (grey) and average across all slices (black) recorded from the perisomatic (A) and dendritic (B) regions of the medial entorhinal cortex, CA3 and CA1. (C) Asymmetry index (see Methods) for the perisomatic and dendritic regions of the three areas. Zero would correspond to a symmetric waveform.

To further explore differences in the gamma oscillations between the different layers, gamma cycle averages were calculated from recordings taken in both the perisomatic and dendritic layers (Fig. 6). From the average gamma cycle waveforms for each condition, the ascending phase was defined as the time between the lowest and the highest value and the descending phase was defined as the time from the highest to the lowest value in the waveforms. In each area, the gamma oscillations recorded in the perisomatic and dendritic layers showed a robust temporal asymmetry with a steep phase going up in the perisomatic layers and down in the dendritic layer followed by a slower phase finishing the cycle (Fig. 6A and B). An asymmetry index between −1 and 1, as defined in the Methods section, was calculated, with zero indicating a symmetrical waveform. For the perisomatic layers, this index was positive, indicating a steeper ascending phase (mEC: 0.25 ± 0.02, *n* = 34 slices; CA3: 0.31 ± 0.05, *n* = 14 slices; CA1: 0.30 ± 0.02, *n* = 39 slices; Fig. 6A and C), while it was negative for the dendritic layers, indicating a steeper descending phase (mEC: −0.25 ± 0.03; CA3: −0.30 ± 0.04; CA1: −0.15 ± 0.02; Fig. 6B and C). Interestingly, independently of the absolute frequency of the gamma oscillations, the asymmetry ratios were similar in all three areas, suggesting perhaps a common mechanism underlying their generation. The reversal in polarity between the different layers was also obvious in the average waveforms, consistent with the CSD analysis (Fig. 6A and B). Furthermore, the CA1 and mEC waveforms appeared to have more of a sawtooth shape than that of CA3 gamma waveforms, which is consistent with reports of CA1 and CA3 gamma oscillation waveforms *in vivo* (Colgin *et al*., 2009; Hsiao *et al*., 2016). Gamma oscillations in the entorhinal–hippocampal system therefore have a consistent asymmetric waveform reflecting the origin of the signal in the perisomatic or dendritic layer of the region.

### Pharmacological analysis of optogenetic entorhinal–hippocampal gamma oscillations

To elucidate the different synaptic components of the gamma generators in the three regions, the pharmacological properties of the three generators were examined. Experiments were conducted by taking a baseline reading, applying the drug and then inducing the gamma oscillations again after a 10‐min period. All three regions showed a small rundown in the power of the gamma oscillations between these two time points even when no drug was added (mEC: 11 ± 2%, *n* = 18, *P* = 0.57; CA3: 6 ± 4%, *n* = 8, *P* = 0.82; CA1: 9 ± 1%, *n* = 19, *P* = 0.0005). All changes in power were therefore compared to this control change when no drug was added. Two of the pharmacological compounds used (bicuculline and NBQX) were solubilised in DMSO. Control application of DMSO in each region had no significant effect on gamma oscillation power (mEC: 85 ± 4%, *n* = 7, *P* = 0.32; CA3: 89 ± 12%, *n* = 6, *P* = 0.61; CA1: 93 ± 3%, *n* = 6, *P* = 0.68).

**Figure 7:**
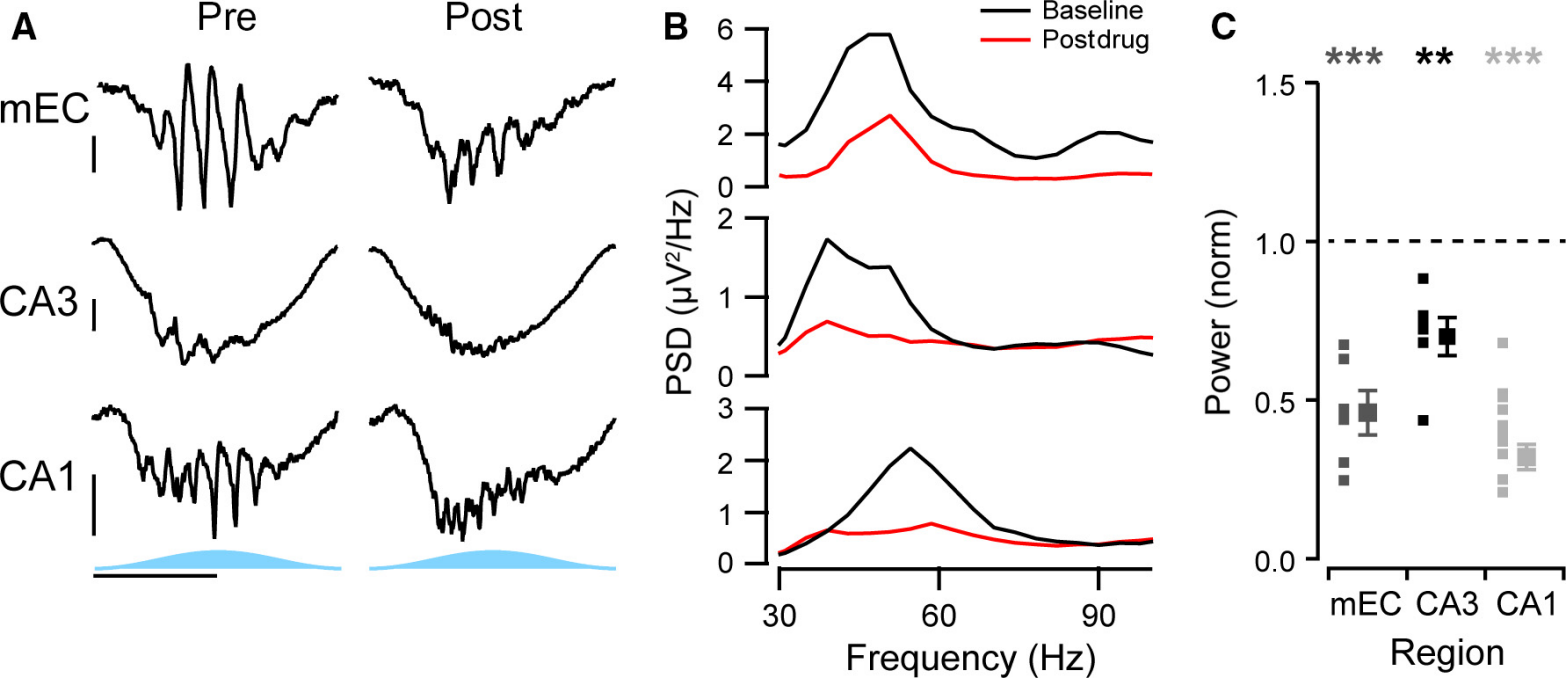
Importance of AMPA/kainate receptors for gamma oscillations. (A) Representative activity of optogenetic gamma oscillations before (left column) and after application of 20 μm NBQX (right column) recorded from the perisomatic region of medial entorhinal cortex (mEC, top row), the dendritic region of CA3 (middle row) and from the perisomatic region of CA1 (bottom row). Horizontal scale bar, 100 ms; vertical scale bar, 50 μV. (B) Power spectral density of the gamma range for the examples given in A, band‐pass filtered between 20 and 120 Hz, before (black line) and after drug treatment (red line). (C) Average change in the power of the gamma oscillations before and after drug, normalised to the change in power in control experiment with no drug added. Small squares represent each individual slice recorded from, and large squares represent the average (*n* = 6, 6 and 14 for the mEC, CA3 and CA1, respectively). Error bars represent standard error of the mean. ***P < 0.001 and **P < 0.005 using an independent samples *t*‐test, respectively.

**Figure 8:**
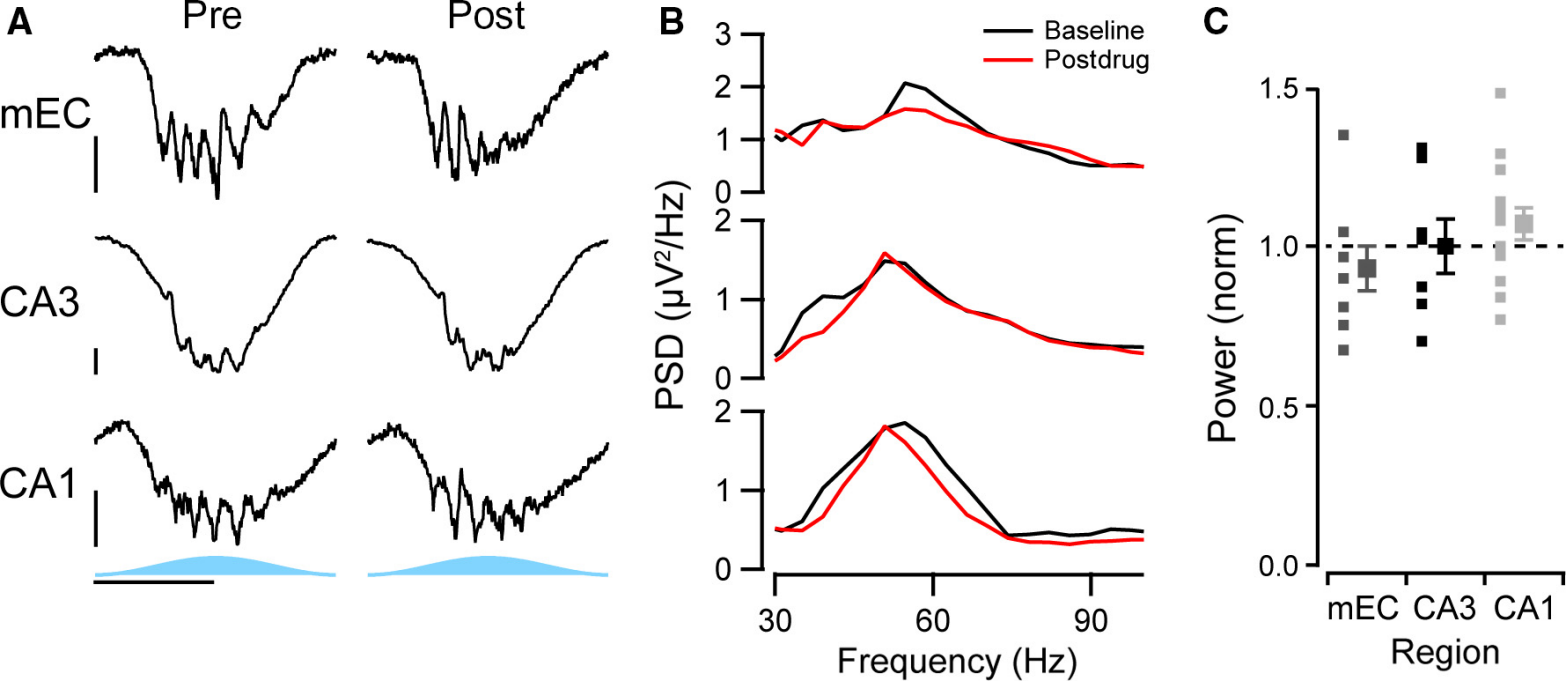
Lack of effect of an NMDA receptor antagonist on gamma oscillations. (A) Representative activity of optogenetic gamma oscillations before (left column) and after application of 50 μm AP5 (right column) recorded from the perisomatic region of medial entorhinal cortex (mEC, top row), dendritic region of CA3 (middle row) and from the perisomatic region of CA1 (bottom row). Horizontal scale bar, 100 ms; vertical scale bar, 50 μV. (B) Power spectral density of the gamma range for the examples given in A, band‐pass filtered between 20 and 120 Hz, before (black line) and after drug treatment (red line). (C) Average change in the power of the gamma oscillations before and after drug, normalised to the change in power in control experiment with no drug added. Small squares represent each individual slice recorded from, and large squares represent the average (*n* = 8, 7 and 13 for the mEC, CA3 and CA1, respectively). Error bars represent standard error of the mean.

To test for the importance of fast glutamatergic excitation, we first blocked AMPA and kainate receptors with 20 μm NBQX (Fig. 7). This significantly decreased the power of the gamma oscillations in all three regions (mEC: 41 ± 6%, *n* = 6, *P* < 0.0001; CA3: 66 ± 6%, *n* = 6, *P* = 0.001; CA1: 37 ± 3%, *n* = 14, *P* < 0.0001; Fig. 7A–C). In contrast, when NMDA receptors were blocked with 50 μm AP5, there was no significant effect on the gamma oscillation power in any of the regions (mEC: 83 ± 7%, *n* = 8, *P* = 0.25; CA3: 95 ± 8%, *n* = 7, *P* = 0.99; CA1: 97 ± 5%, *n* = 13, *P* = 0.21; Fig. 8A–C).

**Figure 9:**
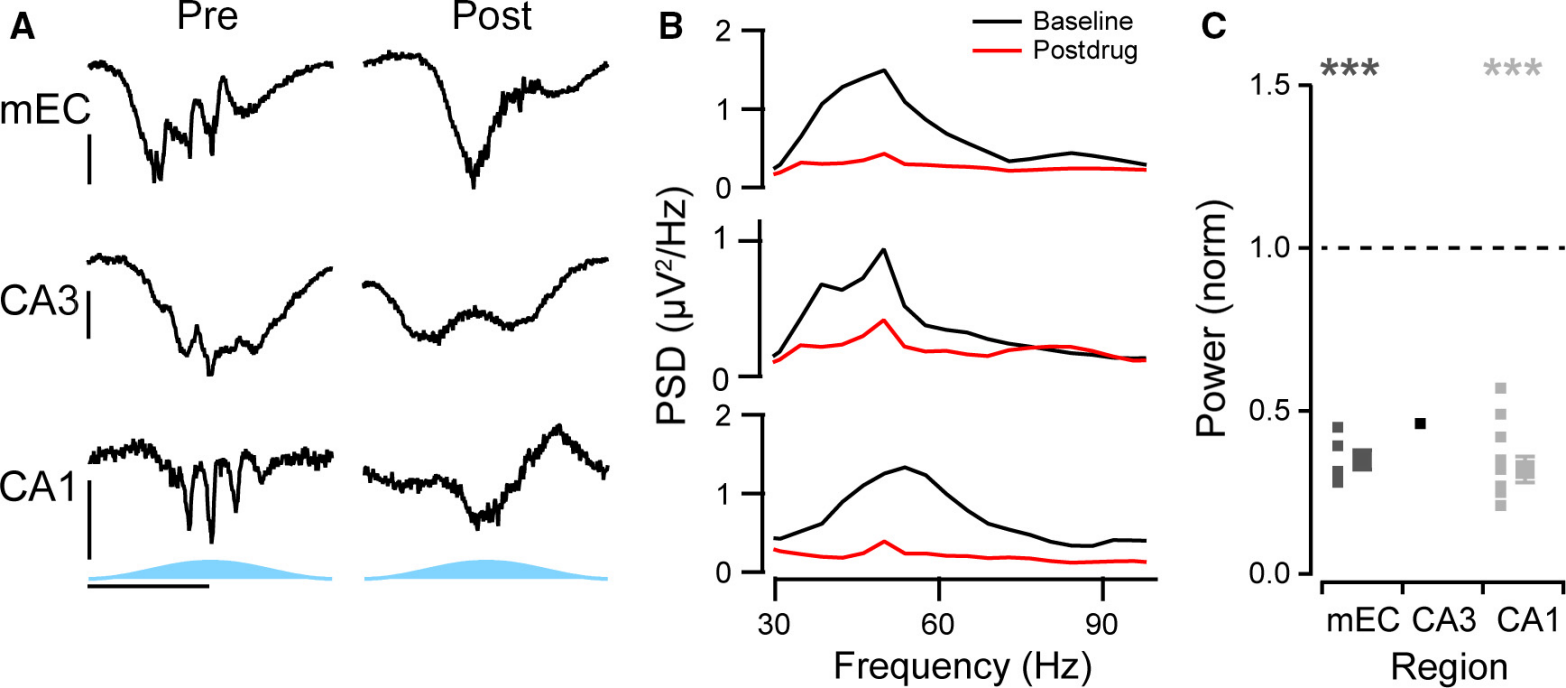
Importance of GABA_A_ receptors for gamma oscillations. (A) Representative activity of optogenetic gamma oscillations before (left column) and after application of 10 μm bicuculline (right column) recorded from the perisomatic region of medial entorhinal cortex (mEC) (top row), dendritic region of CA3 (middle row) and from the perisomatic region of CA1 (bottom row). Horizontal scale bar, 100 ms; vertical scale bar, 50 μV. (B) Power spectral density of the gamma range for the examples given in A, band‐pass filtered between 20 and 120 Hz, before (black line) and after drug treatment (red line). (C) Average change in the power of the gamma oscillations before and after drug, normalised to the change in power in control experiment with no drug added. Small squares represent each individual slice recorded from, and large squares represent the average (*n* = 5, 1 and 9 for the mEC, CA3 and CA1, respectively). Epileptiform activity in CA3 precluded an estimate of gamma power for 3 of 4 slices tested. Error bars represent standard error of the mean. ***P < 0.001 using an independent samples *t*‐test.

The PING mechanism for generating gamma oscillations involves fast GABAergic transmission, and we therefore next blocked GABA_A_ receptors to assess their importance for optogenetically induced gamma oscillations. Bicuculline (10 μm) reduced the power of the gamma oscillations significantly to 31 ± 3% (*n* = 5, *P* < 0.0001) and 32 ± 4% (*n* = 9, *P* < 0.0001) in the mEC and CA1, respectively (Fig. 9A–C). In the case of CA3, network activity shifted to large epileptiform activity in three of four slices tested, probably because of the strong recurrent connections between excitatory neurons in this region (data not shown), which precluded further analysis of their specific involvement in gamma oscillations.

Therefore, the generators underlying the gamma oscillations in the three different regions all required both GABA_A_ and AMPA/kainate receptors, but not NMDA receptors, to function.

## Discussion

By expressing ChR2 in principal neurons of the entorhinal–hippocampal circuit, we have demonstrated that (i) the mEC, CA3 and CA1 can all intrinsically generate gamma oscillations during theta‐rhythmic optogenetic stimulation of principal neurons. (ii) The CA3 gamma oscillations have a lower frequency than the mEC and CA1 optogenetically induced gamma oscillations, consistent with their frequencies *in vivo*. (iii) The asymmetry of gamma oscillation waveforms follows the same pattern across all three regions tested reflecting the layer in which the signals are recorded. (iv) Optogenetically induced gamma oscillations in the mEC, CA3 and CA1 are all pharmacologically similar, depending on both AMPA/kainate and GABA_A_ receptors, but not NMDA receptors.

### Gamma oscillation generators in the entorhinal–hippocampal system

Gamma oscillations in CA1 are heterogeneous, with up to three different oscillations being identified, thought to originate in CA1 and its afferent regions, the mEC and CA3 area (Schomburg *et al*., 2014; Lasztóczi & Klausberger, 2016). By expressing ChR2 in principal neurons in mice, we have shown that slow gamma oscillations can be recorded in CA1 during activation of the CA3 gamma generator *in vivo*, but faster gamma oscillations occur when CA1 itself is stimulated. These properties are shared with spontaneous gamma oscillations *in vivo,* and this suggests that ChR2‐stimulation is a physiologically relevant model of inducing gamma oscillations.

Gamma oscillations could be induced *ex vivo* in all three regions, mEC, CA3 and CA1. This matches previously reported studies of optogenetically induced gamma oscillations in the entorhinal–hippocampal system (Akam *et al*., 2012; Pastoll *et al*., 2013; Butler *et al*., 2016; Betterton *et al*., 2017). The fact that all three regions showed a sink–source pair between the perisomatic and the dendritic layers suggests the presence of an intrinsic current generator in each area.

The ability of each area to generate its own gamma oscillation in the absence of afferent connectivity suggests that these oscillations are not necessarily an inter‐regionally synchronised process, whereby each information packet is bound to one specific gamma cycle which then carries it along the different stages of the hippocampal circuit. Instead, it seems more likely that gamma oscillations have a local processing function, and the downstream region generates a new local gamma oscillation. However, it remains possible that gamma oscillators may be transiently coupled *in vivo*.

### Gamma oscillation frequencies in the entorhinal–hippocampal circuit

Gamma oscillations in the entorhinal–hippocampal system *in vivo* have different frequencies, with the CA3, mEC and CA1 producing what are termed ‘slow’, ‘medium’ and ‘fast’ gamma oscillations, respectively (Belluscio *et al*., 2012; Schomburg *et al*., 2014; Lasztóczi & Klausberger, 2016). In agreement, the CA3 and mEC generated gamma oscillations with ‘slow’ and ‘medium’ frequencies, respectively. However, the frequency of the CA1 gamma oscillations was 66 ± 2 Hz at 30 °C, matching mEC gamma oscillations and below the reported *in vivo* frequency of rat CA1 gamma oscillations of > 100 Hz (Schomburg *et al*., 2014). Unfortunately, we were unable to study gamma oscillations at higher, more physiological temperatures in slices. The frequency of fast gamma oscillations increases with running speed *in vivo* (Zheng *et al*., 2015), which further complicates comparing between *in vivo* and *in vitro* conditions. A recent comprehensive analysis of CA1 gamma oscillations *in vivo* used overlapping boundaries for the medium and fast gamma frequency ranges (60–120 Hz vs. > 100 Hz; Schomburg *et al*., 2014). Therefore, the frequency of the gamma oscillations produced by these two regions may be more similar than originally suggested, and thus, other properties need to be used to distinguish these gamma oscillations, such as their theta‐phase preference. Indeed, the high similarity between medium and fast gamma oscillations offers an explanation as to why Colgin *et al*. (2009) observed gamma oscillations in the medium frequency range occurring at the trough of the theta cycle, which is now thought to be the theta phase at which CA1 gamma oscillations occur (Schomburg *et al*., 2014; Lasztóczi & Klausberger, 2016).

There are several possible explanations for why CA3 produces slower gamma oscillations than the mEC and CA1. As the duration of the inhibitory postsynaptic current (IPSC) is thought to determine the pace of the rhythm (Fisahn *et al*., 1998; Atallah & Scanziani, 2009), it is possible that the generators involve different interneurons which produce IPSCs of different durations thus resulting in gamma oscillations of different frequencies. An alternative explanation is that pyramidal neurons in CA3 have a longer time constant than those in CA1 (Borel *et al*., 2013). Moreover, pyramidal neurons in CA3 are highly recurrently connected (Lorente de Nó, 1934) whereas the principal neurons in CA1 and in layer II of the mEC are not (Pastoll *et al*., 2013). This recurrent excitation may cause a net increase in excitation of interneurons in each gamma cycle compared to networks with less recurrent excitation, thus resulting in larger IPSCs. As the size of IPSCs is positively correlated with the length of each gamma cycle (Atallah & Scanziani, 2009; Butler *et al*., 2016), this would result in a slower gamma frequency for the CA3 region. A fourth possibility is that the synchrony of recurrent excitation is weakened by distributed delays in axonal conduction and/or dendritic integration thus reducing the synchrony of pyramidal cell firing, and consequently extending the time required to activate inhibitory interneurons (Morita *et al*., 2008). These latter hypotheses would explain why the mEC and CA1 both produce similar gamma oscillation frequency and higher than that of CA3 gamma oscillations. Although we cannot completely rule out differential direct recruitment of interneurons by the optogenetic stimulation in the different mouse strains, we find this unlikely as there was similar labelling of PV cells in the two genotypes.

Relatively little is known about whether the observed differences in gamma oscillation frequencies in the entorhinal–hippocampal system have a functional relevance. Recent work has shown place cells to encode different types of information during slow and fast gamma oscillations (Zheng *et al*., 2016), suggesting that the different gamma oscillations serve different functions. However, even on a cycle‐by‐cycle basis, gamma oscillations can vary in frequency by tens of Hz *in vivo* (Atallah & Scanziani, 2009). Therefore, it may be the case that the frequency itself of the gamma oscillations is unimportant, and as long as the timing of principal neuron firing is segregated by the length of a gamma cycle (i.e. anything between 33 and 8 ms), then successful information transfer can occur.

### Gamma oscillation functions in the entorhinal–hippocampal circuit

The communication through coherence hypothesis states that gamma oscillations bidirectionally couple regions with one another so that information arrives in the downstream region in a synchronised window of excitation thus facilitating information transfer (Fries, 2005, 2015). While a direct bidirectional coupling exists between the mEC and CA1, the excitatory connections between CA3 and CA1 appear to be unidirectional. This would mean that, for CA3–CA1 gamma oscillations, an intermediary third region would be needed for bidirectional coupling. In this study gamma oscillations were induced in the absence of afferent activity, meaning that gamma oscillations can at least exist in the absence of such third regions. It would be interesting to see the effect of activating gamma oscillations in two regions simultaneously to see if any bidirectional coupling is observed, especially as this has been suggested to happen to theta oscillations in the entorhinal–hippocampal system (Colgin, 2013).

The waveform of the gamma oscillations in the perisomatic layer was highly consistent between the three regions, with a fast ascending phase that lasted approximately a quarter of a cycle and a slower descending phase that lasted for the remaining three quarters of the cycle. In the dendritic layers, the opposite was true, with a short descending phase and a longer ascending phase. This fast rise and slow decay are reflective of the synaptic currents that underlie the gamma oscillations, but unfortunately does not allow for the distinction between excitatory and inhibitory currents because both possess a fast first phase and slower second phase. Nonetheless, this information could aid in determining which layer an oscillation is being recorded from, based on the lengths of the ascending and descending phases of each gamma cycle.

### Mechanism of generation of gamma oscillations

There was a similar pharmacological profile across the three regions, with the power of all three gamma generators being severely impaired when either excitatory AMPA/kainate receptors or inhibitory GABA_A_ receptors were blocked. In the case of block of AMPA/kainate receptors, small residual gamma oscillations remained (Fig. 7A). This is consistent with previous studies of ChR2‐induced CA1 gamma oscillations (Dine *et al*., 2016), where it was observed that blocking muscarinic acetylcholine receptors with atropine eliminated these residual gamma oscillations. These results indicate that a fast excitatory–inhibitory feedback loop underlies the generation of gamma oscillations during theta‐rhythmic activation of principal cells in all three regions, consistent with a PING mechanism of generation (Fisahn *et al*., 1998; Whittington *et al*., 2000). However, because principal neurons were directly activated in the experiment, this result only shows that a PING mechanism is sufficient for the generation of fast gamma oscillations, and we cannot exclude the possibility that tonic activation of interneurons contributes to theta–gamma oscillations *in vivo*.

## Conflict of interest

The authors declare no competing financial interests.

## Author contributions

J.L.B. and O.P designed experiments; J.L.B. performed and analysed electrophysiology experiments; J.L.B. and Y.A.H. performed and analysed immunohistochemistry experiments; all authors wrote the manuscript and approved the final version.

## Data accessibility

Primary data are available from the authors on request.

**Abbreviations**
AC: alternating current
aCSF: artificial cerebrospinal fluid
AMPA: α‐amino‐3‐hydroxy‐5‐methyl‐4‐isoxazolepropionic acid
AP5: dl‐2‐amino‐5‐phosphonopentanoic acid
AWERB: animal welfare and ethical review body
CA: cornu ammonis
ChR2: channelrhodopsin‐2
CSD: current source density
DMSO: dimethyl sulfoxide
eYFP: enhanced yellow fluorescent protein
GABA: γ‐aminobutyric acid
GFP: green fluorescent protein
ING: interneuron network gamma
IPSC: inhibitory postsynaptic current
LFP: local field potential
MEA: multielectrode array
mEC: medial entorhinal cortex
NBQX: 2,3‐dihydroxy‐6‐nitro‐7‐sulfamoyl‐benzo[f]quinoxaline‐2,3‐dione
NMDA: *N*‐methyl‐d‐aspartate
PBS: phosphate‐buffered saline
PING: pyramidal‐interneuron network gamma
PSD: power spectral density
PV: parvalbumin
