# Supplementary material for: Comparison of three gamma oscillations in the mouse entorhinal–hippocampal system
Source: Eur J Neurosci. 2018 Feb 9;48(8):2795–806. doi: 10.1111/ejn.13831 (PMC6221063; doi:10.1111/ejn.13831)
Supplement: Supplementary file 1 [file EJN-48-2795-s001.pdf]

EJN European Journal of Neuroscience FENS Federation of European Neuroscience Societies

Comparison of three gamma oscillations in the mouse entorhinal-hippocampal system

James L. Butler, Y. Audrey Hay & Ole Paulsen

Review timeline:

Submission date:	22 August 2017
Editorial Decision:	09 October 2017
Revision received:	29 November 2017
Accepted:	08 January 2018

Editor: Heleen Slagter

1st Editorial Decision 09 October 2017

Dear Ole,

Your manuscript has been reviewed by two expert external reviewers as well as by the Section Editor, Dr. Heleen Slagter, and ourselves. The reviews collectively indicate that your experiments generated new and important information. However, there are several issues that need to be clarified/resolved in a revised version of the manuscript before we can consider your manuscript further for publication in EJN.

As you can see, neither of the reviewers had any major concerns about your manuscript, but both raise some careful and constructive comments that may improve the interpretation of the results and the clarity of the presentation. Please carefully consider each of their points. Please also provide a list of abbreviations, an author contributions statement and perhaps define mEC in the abstract.

If you are able to respond fully to the points raised, we would be pleased to receive a revision of your paper within 12 weeks.

Thank you for submitting your work to EJN and support of this Special Issue.

Best wishes,

Paul & John
co-Editors in Chief, EJN

Reviews:

Reviewer: 1 (Laura Colgin, The University of Texas at Austin, USA)

Comments to the Author
This is a very important and well-written manuscript that combines optogenetics with in vivo and in vitro recordings to elucidate mechanisms of theta-nested slow and fast gamma rhythms in the entorhinal-hippocampal network. I have no major concerns about this manuscript, but I do have a number of recommendations, listed below:

1. When discussing interpretations of their results, the authors may also want to consider the possibility that each region generates oscillations independently, as the authors mention, but that the oscillators become coupled, which could facilitate the transfer of information, as others have suggested. A similar framework has previously been put forward for theta rhythms in different regions.
2. In the pharmacology experiments, was there also a washout period of recording?
3. In the Methods, the authors describe how they constructed the current source density profiles. Is there a citation for a previous use of this method with these probes? If so, it should perhaps be cited.
4. On pages 9-10, the authors write, “As is the case with gamma oscillations recorded in vivo, the oscillations recorded here were all phase-amplitude coupled to the theta stimulation”, but references are missing after this statement. Theta-gamma coupling in mice has been shown by the Klausberger Lab and in

Mably et al., Hippocampus 2017.
5. When discussing CA3 slow gamma oscillations, the authors should also cite the slow gamma oscillations that have been described in CA3 and the dentate gyrus in freely behaving rats (Hsiao et al., J Neurophys 2016). For example, this reference should be included after “getting closer to the in vivo frequency of slow gamma” on page 10.
6. On page 10, the authors write, “The frequency of gamma oscillations in the mEC at 30 degrees C were also in agreement with the medium gamma oscillations described in vivo, reaching a frequency of 68 +- 3 Hz”. References are missing after this statement. Zheng et al., Hippocampus 2015 could be cited here for gamma oscillations in this frequency range in mEC.
7. On page 10, the authors write, “Gamma oscillations in CA1 were slower than the fast gamma oscillations seen in vivo, reaching a frequency of 66 +- 2 Hz.” Yet, this frequency is consistent with gamma oscillations reported in CA1 in mice (Mably et al., Hippocampus 2017). It may also be important to note here that fast gamma frequencies in CA1 increase with movement speed (Zheng et al., Hippocampus 2015) and that may be related to the differences in the present study and other in vivo reports.
8. On page 11, the authors write, “The frequency of all three gamma oscillations remained stable, however, with CA3 gamma oscillations consistently slower than mEC or CA1 gamma oscillations across all stimulation intensities tested”. Yet, the authors did not appear to run a statistical test to determine whether frequency changed with stimulation intensity, and they do not discuss frequency changes with stimulation intensity. It appears as though 100% stimulation for MEC may have induced faster gamma than 60% intensity and that 100% stimulation intensity for CA3 may have induced slower gamma than 60% intensity, but this is not mentioned.
9. Related to the above point, in the quoted sentence from page 11, I recommend that the authors change “The frequency of all three gamma oscillations” to “The frequency relationships between all three gamma oscillations”.
10. On page 12, the authors state that “the waveforms of the gamma oscillations were remarkably similar between mEC, CA3, and CA1, indicating a common mechanism underlying their generation”. I didn’t really agree with this part- to me, the waveforms appear to have more of a sawtooth shape in CA1 and mEC compared to the CA3 waveform, and this is similar to gamma oscillations in vivo (e.g., see CA1 and CA3 gamma waveforms in Colgin et al., 2009, Hsiao et al., 2016, etc.). This sawtooth shape is also apparent in the CA1 recordings shown in Figure 5B (right).
11. Related to the above point, I didn’t really understand how the symmetry of the waveforms was measured. The steep phase of the average CA1 waveform in Figure 6A (right panel) appears to me to be about 40 degrees shorter than the steep phase duration from the CA3 recordings, unless I am misunderstanding something. What exactly is defined as the ascending and descending phase in these waveforms?
12. On pages 12-13, the authors write at the top of the page, “There was no significant change in the frequencies between the two time points”. Do they mean the timepoints with the drug or without the drug?
13. In the beginning of the Discussion on page 13, the authors write, “gamma oscillations in the mEC, CA3, and CA1 are all broadly similar”, but this is not really backed up by the data. The waveforms look different, and CA1 sink/sources were only seen in s.l.m., not s. radiatum. They could say that the oscillations were pharmacologically similar, which is a statement backed up by their data.
14. On page 15, the authors state, “Relatively little is known about whether the observed differences in gamma oscillation frequencies in the entorhinal-hippocampal system have a functional relevance”. However, place cells have been shown to code information differently during slow and fast gamma (Zheng et al., Neuron 2016).
15. The Figure 3 panels D-G are incorrectly described in the legend (e.g., the y-axis in panel D is labeled as “phase” but described as power in the legend).
16. In the Figure 4B legend, I recommend changing “Power spectral density of the recordings” to “Power spectral density of the filtered recordings”.
17. The CA3 post-drug data are not shown in Figure 9B, but couldn’t the authors choose the example slice in which epileptiform activity was not generated? Likewise, couldn’t this single data point be included in Figure 9C?
18. The time ticks on the x-axis in Figure 2A are strange. Why not 100 ms tick marks so that readers can more easily count cycles and discern frequency?
19. The authors may want to consider changing the shape of the power spectrum plots in Figure 2C (i.e., square axes) and plotting CA3 and CA1 on top of each other for ease of comparison.
20. In Figure 4A, it appears as though the theta phase at which gamma maximally occurred also changed with stimulation intensity. If so, that is interesting. Was that the case or does it just seem that way in these examples?
21. In Figure 7A (bottom right), it appears as though faster oscillations emerged in CA1 when AMPA/kainite receptors were blocked. Was that observed in all cases or just this example? If it was the former, that is potentially interesting and perhaps should be mentioned.

Reviewer: 2 (Dimitri Kullman, University College London, UK)

Comments to the Author
The manuscript by Butler et al provides an interesting comparison of gamma oscillations elicited by theta-

EJN European Journal of Neuroscience FENS Federation of European Neuroscience Societies

modulated optogenetic drive applied to CA1, CA3 and EC. Some in vivo data are shown, but most of the analysis is derived from in vitro slice experiments. In keeping with the literature, the frequency of gamma is lower in CA3, although the authors do not find differences between CA1 and EC. The mechanisms underlying the difference in frequency between CA3 and CA1/EC remain to be determined, although the authors speculate about the possible roles of recurrent connections in CA3, interneuron connectivity and IPSC kinetics. Another possibility is that CA3 pyramidal neurons themselves have longer membrane time constants (Neuroelectro.org).

There are no major surprises in the data, and the presentation of the results is clear. A few issues should be addressed:
1. The design of the ‘optrode’ for in vivo experiments is not clear: what was the diameter of the optical fiber? Where was the electrode tip in relation to the optical fiber? How did the authors deal with possible photoelectric artefacts?
2. P. 10 line 3: “...occurred near the peak of the theta...” Should this be trough?
3. Fig. 9 and text: the residual gamma in bicuculline is not convincing. Indeed, it is incompatible with either PING or ING. Were GABAA receptors fully blocked?

Authors’ Response 29 November 2017

Reviewer: 1

This is a very important and well-written manuscript that combines optogenetics with *in vivo* and *in vitro* recordings to elucidate mechanisms of theta-nested slow and fast gamma rhythms in the entorhinal-hippocampal network. I have no major concerns about this manuscript, but I do have a number of recommendations, listed below:

We would like to thank the Reviewer for their positive assessment of our manuscript. We have dealt with the recommendations as outlined below. The corresponding changes in the manuscript have been highlighted in yellow.

1. **When discussing interpretations of their results, the authors may also want to consider the possibility that each region generates oscillations independently, as the authors mention, but that the oscillators become coupled, which could facilitate the transfer of information, as others have suggested. A similar framework has previously been put forward for theta rhythms in different regions.**

This is a good point, which we failed to consider in our manuscript. We have now updated the discussion to mention this possibility (page 15 and 16):

(Page 15) However, it remains possible that gamma oscillators may be transiently coupled in vivo.

(Page 16/17) In this study gamma oscillations were induced in the absence of afferent activity, meaning that gamma oscillations can at least exist in the absence of such third regions. It would be interesting to see the effect of activating the gamma oscillations in two regions simultaneously to see if any bidirectional coupling is observed, especially as this has been suggested to happen to theta oscillations in the entorhinal-hippocampal system (Colgin, 2013).

2. **In the pharmacology experiments, was there also a washout period of recording?**

No washouts were done during recording. However, obviously, in between each slice recording, the rig was superfused thoroughly with water to remove any residual traces of the pharmacological agents from the set up.

3. **In the Methods, the authors describe how they constructed the current source density profiles. Is there a citation for a previous use of this method with these probes? If so, it should perhaps be cited.**

We have updated the text with citations of previous uses of this technique. The CSD section is now prefaced with the following sentence (page 8):

A current source density (CSD) profile was constructed from signals recorded on the 64-channel array as described previously (Mann et al., 2005; Butler et al., 2016).

4. **On pages 9-10, the authors write, "As is the case with gamma oscillations recorded *in vivo*, the oscillations recorded here were all phase-amplitude coupled to the theta stimulation", but references are missing after this statement. Theta-gamma coupling in mice has been shown by the Klausberger Lab and in Mably *et al.*, Hippocampus 2017.**

We have updated the text to include seminal citations that gamma oscillations are phase-amplitude coupled to theta oscillations (page 10). These references have also been added to the reference list.

As is the case with gamma oscillations recorded in vivo (Bragin et al., 1995; Csicsvari et al., 2003; Colgin et al., 2009), the gamma oscillations recorded here were all phase-amplitude coupled to the theta oscillation, having the highest power at the trough of the theta LFP as recorded in the perisomatic region (Figure 3C).

5. **When discussing CA3 slow gamma oscillations, the authors should also cite the slow gamma oscillations that have been described in CA3 and the dentate gyrus in freely behaving rats (Hsiao *et al.*, J Neurophys 2016). For example, this reference should be included after "getting closer to the *in vivo* frequency of slow gamma" on page 10.**

We have updated the text to include the suggested citation on page 10:

For CA3, gamma oscillations increased from 23 ± 0 Hz at 22 °C to 41 ± 2 Hz at 30 °C (n = 14; Figure 3H), getting closer to the in vivo slow gamma frequency (Schomburg et al., 2014; Hsiao et al., 2016; Lasztóczi & Klausberger, 2016).

6. **On page 10, the authors write, "The frequency of gamma oscillations in the mEC at 30 degrees C were also in agreement with the medium gamma oscillations described *in vivo*, reaching a frequency of 68 +- 3 Hz". References are missing after this statement. Zheng *et al.*, Hippocampus 2015 could be cited here for gamma oscillations in this frequency range in mEC.**

We have updated the text to include citations for medium gamma frequency *in vivo* (page 10):

Gamma oscillations in CA1 had a frequency of 66 ± 2 Hz at 30 °C (n = 12; Figure 3H), slower than fast gamma frequency recorded in rats in vivo (Colgin et al., 2009; Belluscio et al., 2012, Schomburg et al., 2014) but comparable to fast gamma oscillations in mice (Mably et al., 2017).

7. **On page 10, the authors write, "Gamma oscillations in CA1 were slower than the fast gamma oscillations seen *in vivo*, reaching a frequency of 66 +- 2 Hz." Yet, this frequency is consistent with gamma oscillations reported in CA1 in mice (Mably *et al.*, Hippocampus 2017). It may also be important to note here that fast gamma frequencies in CA1 increase with movement speed (Zheng *et al.*, Hippocampus 2015) and that may be related to the differences in the present study and other *in vivo* reports.**

We updated the results section to comment how this matches the frequency of mouse gamma oscillations (page 10):

Gamma oscillations in CA1 had a frequency of 66 ± 2 Hz at 30 °C (n = 12; Figure 3H), slower than fast gamma frequency recorded in rats in vivo (Colgin et al., 2009; Belluscio et al., 2012, Schomburg et al., 2014) but comparable to fast gamma oscillations in mice (Mably et al., 2017).

EJN European Journal of Neuroscience FENS Federation of European Neuroscience Societies

We now also mention in the discussion the fact that frequency varies with running speed, complicating comparison of *in vitro* and *in vivo* studies (page 15):

Unfortunately, we were unable to study gamma oscillations at higher, more physiological temperatures in slices. The frequency of fast gamma oscillations increases with running speed in vivo (Zheng et al., 2015), which further complicates comparing between in vivo and in vitro conditions. A recent comprehensive analysis of CA1 gamma oscillations in vivo used overlapping boundaries for the medium and fast gamma frequency ranges (60 – 120 Hz vs >100 Hz; Schomburg et al., 2014).

8. **On page 11, the authors write, "The frequency of all three gamma oscillations remained stable, however, with CA3 gamma oscillations consistently slower than mEC or CA1 gamma oscillations across all stimulation intensities tested". Yet, the authors did not appear to run a statistical test to determine whether frequency changed with stimulation intensity, and they do not discuss frequency changes with stimulation intensity. It appears as though 100% stimulation for MEC may have induced faster gamma than 60% intensity and that 100% stimulation intensity for CA3 may have induced slower gamma than 60% intensity, but this is not mentioned.**

Stimulation intensity did significantly increase CA1 gamma frequency, but there was no significant effect on CA3 or mEC intensity. We have updated the text to provide a more accurate and thorough description of the effect of light intensity on gamma frequency (page 11):

Frequency could not be accurately estimated at 40% maximum light intensity due to the lower power of gamma oscillations induced; we therefore compared the frequency at 60%, 80% and 100% of maximum light intensity. CA1 gamma oscillation frequency increased significantly from 61 ± 1 Hz to 70 ± 2 Hz (p = 0.004, Figure 4D) when the light intensity was increased from 60% to 100% of maximum intensity. The frequency of mEC and CA3 gamma oscillations, however, was not significantly different between these two light intensities (59 ± 6 Hz and 63 ± 5 Hz, p = 0.35, for the mEC, 49 ± 1 Hz and 45 ± 2 Hz, p = 0.10, for CA3, Figure 4D).

9. **Related to the above point, in the quoted sentence from page 11, I recommend that the authors change "The frequency of all three gamma oscillations" to "The frequency relationships between all three gamma oscillations".**

This sentence has been removed (see the response to point 8).

10. **On page 12, the authors state that "the waveforms of the gamma oscillations were remarkably similar between mEC, CA3, and CA1, indicating a common mechanism underlying their generation". I didn't really agree with this part- to me, the waveforms appear to have more of a sawtooth shape in CA1 and mEC compared to the CA3 waveform, and this is similar to gamma oscillations *in vivo* (e.g., see CA1 and CA3 gamma waveforms in Colgin *et al.*, 2009, Hsiao *et al.*, 2016, etc.). This sawtooth shape is also apparent in the CA1 recordings shown in Figure 5B (right).**

We agree and have updated the results text to include this observation (page 13):

The reversal in polarity between the different layers was also obvious in the average waveforms, consistent with the CSD analysis (Figure 6A, B). Furthermore, the CA1 and mEC waveforms appeared to have more of a sawtooth shape than that of CA3 gamma waveforms, which is consistent with reports of CA1 and CA3 gamma oscillation waveforms in vivo (Colgin et al., 2009; Hsiao et al., 2016).

11. **Related to the above point, I didn't really understand how the symmetry of the waveforms was measured. The steep phase of the average CA1 waveform in Figure 6A (right panel) appears to me to be about 40 degrees shorter than the steep phase**

duration from the CA3 recordings, unless I am misunderstanding something. What exactly is defined as the ascending and descending phase in these waveforms?

The ascending phase was defined as the time between the lowest value and the highest value in the average waveform, while the descending phase was defined as the time from the lowest to the highest value in the average waveforms. We are sorry that our original analysis was not clear. We have therefore replaced the analysis with an asymmetry index similar to that used by Belluscio *et al.*, 2012. The asymmetry index was calculated as the duration of the descending phase as a fraction of a half cycle minus 1. This index produces a value of 0 for a perfectly symmetric waveform, a value between 0 and 1 for waveforms with a shorter ascending phase (with 1 being if the descending phase occupied the entire waveform) and between 0 and -1 for waveforms with a longer ascending phase.

While the peak of the perisomatic gamma cycle in CA1 occurs later than in CA3 in the graph in 6A, this is also true of the trough of the perisomatic gamma cycle for CA1. Measuring the time between the peak and the trough therefore results in a longer steep phase for CA1 compared to CA3. We have updated the text to indicate how this index was calculated and the values it produced:

Methods (page 8):

To characterise the temporal waveform of the oscillation, an asymmetry index was defined as the duration of the descending phase as a fraction of a half cycle minus 1. This index produces a value of 0 for a perfectly symmetric waveform, a value between 0 and 1 for waveforms with a shorter ascending phase (with 1 being if the descending phase occupied the entire waveform) and between 0 and -1 for waveforms with a longer ascending phase.

Results (page 12):

To further explore differences in the gamma oscillations between the different layers, gamma cycle averages were calculated from recordings taken in both the perisomatic and dendritic layers (Figure 6). From the average gamma cycle waveforms for each condition, the ascending phase was defined as the time between the lowest and the highest value and the descending phase was defined as the time from the highest to the lowest value in the waveforms. In each area, the gamma oscillations recorded in the perisomatic and dendritic layers showed a robust temporal asymmetry with a steep phase going up in the perisomatic layers and down in the dendritic layer followed by a slower phase finishing the cycle (Figure 6A, B). An asymmetry index between -1 and 1, as defined in the Methods section, was calculated, with zero indicating a symmetrical waveform. For the perisomatic layers this index was positive, indicating a steeper ascending phase (mEC: 0.25 ± 0.02, n = 34 slices; CA3: 0.31 ± 0.05, n = 14 slices; CA1: 0.30 ± 0.02, n = 39 slices; Figure 6A, C) while it was negative for the dendritic layers, indicating a steeper descending phase (mEC: -0.25 ± 0.03; CA3: -0.30 ± 0.04; CA1: -0.15 ± 0.02; Figure 6B, C). Interestingly, independently of the absolute frequency of the gamma oscillations, the asymmetry ratios were similar in all three areas, suggesting perhaps a common mechanism underlying their generation.

12. On pages 12-13, the authors write at the top of the page, “There was no significant change in the frequencies between the two time points”. Do they mean the timepoints with the drug or without the drug?

Because it was difficult to accurately estimate the peak frequency at low gamma powers (see Point 8), we no longer report on changes in frequency in the pharmacology part of the manuscript.

13. In the beginning of the Discussion on page 13, the authors write, “gamma oscillations in the mEC, CA3, and CA1 are all broadly similar”, but this is not really backed up by the data. The waveforms look different, and CA1 sink/sources were only seen in s.l.m., not s. radiatum. They could say that the oscillations were pharmacologically similar, which is a statement backed up by their data.

We have amended the text to make this suggested correction (page 14):

4) Optogenetically-induced gamma oscillations in the mEC, CA3 and CA1 are all pharmacologically similar, depending on both AMPA/kainate and $GABA_A$ receptors, but not NMDA receptors.

14. On page 15, the authors state, "Relatively little is known about whether the observed differences in gamma oscillation frequencies in the entorhinal-hippocampal system have a functional relevance". However, place cells have been shown to code information differently during slow and fast gamma (Zheng *et al.*, Neuron 2016).

We have updated the text to mention this fact (page 16):

Relatively little is known about whether the observed differences in gamma oscillation frequencies in the entorhinal-hippocampal system have a functional relevance. Recent work has shown place cells to encode different types of information during slow and fast gamma oscillations (Zheng et al., 2016) suggesting that the different gamma oscillations serve different functions. However, even on a cycle-by-cycle basis gamma oscillations can vary in frequency by tens of Hz in vivo (Atallah & Scanziani, 2009). Therefore, it may even be the case that the frequency itself of the gamma oscillations is not important, and as long as the timing of principal neuron firing is segregated by the length of a gamma cycle (i.e. anything between 33 ms and 8 ms), then successful information transfer can occur.

15. The Figure 3 panels D-G are incorrectly described in the legend (e.g., the y-axis in panel D is labeled as "phase" but described as power in the legend).

This has now been corrected (page 21):

D. *Average phase of the theta oscillation at which the peak gamma oscillation occurred in each of the three regions. 0° corresponds to the peak of the theta cycle.* ***E.*** *Average power of the gamma oscillations in the three different regions.* ***F.*** *Average ratio of the gamma power to the theta power elicited by stimulation.* ***G.*** *Average peak frequency of gamma oscillations in the three different regions.*

16. In the Figure 4B legend, I recommend changing "Power spectral density of the recordings" to "Power spectral density of the filtered recordings".

We have amended the Figure legend as suggested (page 22):

B. *Power spectral density of the filtered recordings shown in A.*

17. The CA3 post-drug data are not shown in Figure 9B, but couldn't the authors choose the example slice in which epileptiform activity was not generated? Likewise, couldn't this single data point be included in Figure 9C?

We have now altered the figure to show the example slice without epileptiform activity, and included this data point in panel 9C.

18. The time ticks on the x-axis in Figure 2A are strange. Why not 100 ms tick marks so that readers can more easily count cycles and discern frequency?

The ticks have been amended to 100 ms intervals, rather than 83 ms intervals.

19. The authors may want to consider changing the shape of the power spectrum plots in Figure 2C (i.e., square axes) and plotting CA3 and CA1 on top of each other for ease of comparison.

We have now made this change and plotted the two power spectrums on top of one another.

20. **In Figure 4A, it appears as though the theta phase at which gamma maximally occurred also changed with stimulation intensity. If so, that is interesting. Was that the case or does it just seem that way in these examples?**

We did not look at changes in the theta phase of gamma oscillations occurring as the theta-stimulation is artificial and so we are not sure of the relevance of any findings. If we had induced theta oscillations with a more physiological method, such as stimulation of medial septum inputs to the hippocampus, then this would certainly be something interesting to look at.

21. **In Figure 7A (bottom right), it appears as though faster oscillations emerged in CA1 when AMPA/kainite receptors were blocked. Was that observed in all cases or just this example? If it was the former, that is potentially interesting and perhaps should be mentioned.**

Small oscillations remained after AMPA/Kainate receptor blockade. This is consistent with the findings of Dine *et al.* (2016, Front. Cell. Neuro.) who found a similar effect when blocking excitatory receptors during ChR2-induced CA1 gamma oscillations. The authors also showed that blocking acetylcholine receptors with atropine got rid of these residual oscillations, demonstrating a small contribution of ACh receptors for the oscillations. We did not investigate this question further as the low signal-to-noise ratio made it very difficult to analyse these residual oscillations and we could not confidently isolate these small high frequency oscillations from theta oscillation harmonics. We have updated the discussion to mention this (page 17):

There was a similar pharmacological profile across the three regions, with the power of all three gamma generators being severely impaired when either excitatory AMPA/kainate receptors or inhibitory $GABA_A$ receptors were blocked. In the case of block of AMPA/kainate receptors small residual gamma oscillations remained (Figure 7A). This is consistent with previous studies of ChR2-induced CA1 gamma oscillations (Dine *et al.*, 2016), where it was observed that blocking muscarinic acetylcholine receptors with atropine eliminated these residual gamma oscillations.

Reviewer: 2

The manuscript by Butler et al provides an interesting comparison of gamma oscillations elicited by theta-modulated optogenetic drive applied to CA1, CA3 and EC. Some *in vivo* data are shown, but most of the analysis is derived from *in vitro* slice experiments. In keeping with the literature, the frequency of gamma is lower in CA3, although the authors do not find differences between CA1 and EC. The mechanisms underlying the difference in frequency between CA3 and CA1/EC remain to be determined, although the authors speculate about the possible roles of recurrent connections in CA3, interneuron connectivity and IPSC kinetics. Another possibility is that CA3 pyramidal neurons themselves have longer membrane time constants (Neuroelectro.org).

We would like to thank the Reviewer for their positive assessment of our manuscript. We have added the possibility that CA3 pyramidal neurons have longer membrane time constant (page 15).

An alternative explanation is that pyramidal neurons in CA3 have a longer time constant than those in CA1 (Borel et al., 2013).

1. **The design of the 'optrode' for *in vivo* experiments is not clear: what was the diameter of the optical fiber? Where was the electrode tip in relation to the optical fiber? How did the authors deal with possible photoelectric artefacts?**

No photovoltaic artifacts were observed during recording. Also, as we were stimulating at theta frequency photovoltaic artifacts in the gamma frequency range are unlikely. We agree this would be a problem if we had wanted to calculate theta power. We have updated the methods section with details of the fibre optic setup (page 5):

To record the local field potential (LFP) in vivo, a 2 MΩ tungsten recording electrode (A-M systems, Hinckley, United Kingdom (UK)) was attached to a 200 µm diameter optical fibre (Thorlabs, New Jersey, USA) with the electrode tip protruding approximately 100 µm past the tip of the optical fibre.

2. P. 10 line 3: "…occurred near the peak of the theta…" Should this be trough?

The Reviewer is correct; this has been amended in the text:

On average, all three gamma oscillations occurred near the trough of the theta LFP oscillations.

3. Fig. 9 and text: the residual gamma in bicuculline is not convincing. Indeed, it is incompatible with either PING or ING. Were GABAA receptors fully blocked?

It is possible that we did not achieve full blockade of $GABA_A$ receptors as we performed no functional test of their activity during bicuculline application. The effect of pharmacological compounds on the gamma oscillations was calculated as the change in the area under the PSD ± 15 Hz from the peak in the gamma range. A limitation of this method is that it does not account for any noise in the baseline spectral power. Thus, even if the gamma oscillations are completely abolished but the noise is not (as is expected), then there will be residual noise power included in the gamma power estimate. This may therefore result in an underestimation of the effect of the pharmacological compounds on the power of the gamma oscillations. However, rather than attempting baseline subtraction, we believe this is the most robust and unbiased way of estimating gamma power.

We would like to thank the Reviewers again for their constructive comments, which have helped improve the paper, and we trust it is now acceptable for publication in the EJN.
